# Nutrition and Cardiovascular Disease: Finding the Perfect Recipe for Cardiovascular Health

**DOI:** 10.3390/nu8060363

**Published:** 2016-06-14

**Authors:** Alice Ravera, Valentina Carubelli, Edoardo Sciatti, Ivano Bonadei, Elio Gorga, Dario Cani, Enrico Vizzardi, Marco Metra, Carlo Lombardi

**Affiliations:** Division of Cardiology, Department of Medical and Surgical Specialties, Radiological Sciences, and Public Health, University and Civil Hospital of Brescia, Brescia 25123, Italy; ravera.alice@gmail.com (A.R.); valentina.carubelli@gmail.com (V.C.); edoardo.sc@tin.it (E.S.); ivano.bonadei@libero.it (I.B.); eliogorga@gmail.com (E.G.); canidarios@gmail.com (D.C.); isotta5@hotmail.com (E.V.); metramarco@libero.it (M.M.)

**Keywords:** diet, Mediterranean, DASH, cardiovascular disease, coronary artery disease, heart failure, hypertension

## Abstract

The increasing burden of cardiovascular disease (CVD) despite the progress in management entails the need of more effective preventive and curative strategies. As dietary-associated risk is the most important behavioral factor influencing global health, it appears the best target in the challenge against CVD. Although for many years, since the formulation of the *cholesterol hypothesis*, a nutrient-based approach was attempted for CVD prevention and treatment, in recent years a dietary-based approach resulted more effective in reducing cardiovascular risk worldwide. After the publication of randomized trials on the remarkable effects of the Mediterranean diet and the Dietary Approach to Stop Hypertension (DASH) diet on CVD, new efforts were put on research about the effects of complex dietary interventions on CVD. The purpose of this paper is to review the evidence on dietary interventions in the prevention and disease modification of CVD, focusing on coronary artery disease and heart failure, the main disease responsible for the enormous toll taken by CVD worldwide.

## 1. Introduction

Even though the global burden of cardiovascular disease (CVD) has steadily decreased during the past 10 years, CVD remains the leading cause of death and disability in developed countries. In fact, CVD is responsible for approximately one of every three deaths in the United States and one of every four deaths in Europe [1,2]. Moreover, developing countries underwent a steep increase in the incidence of CVD over the last 25 years, now being the second cause of years of life lost in most of these countries [3], due in part to their acquisition of Western patterns of diet [4]. The substantial magnitude of the global burden of CVD, despite the progress made in therapy, underscores the need of effective strategies to prevent and modify the course of this widespread disease. Explaining about one-third of global mortality, dietary risk appears to be a priority target for CVD prevention and treatment [3]. The purpose of this paper is to review the evidence on dietary interventions in the prevention and disease modification of CVD, focusing on the most widespread CVD: coronary artery disease (CAD) and heart failure (HF). The authors acknowledge the importance of single nutrients and their role in CVD, however extensive review of their role in CAD and HF is already present in the literature and goes beyond the aim of this paper [5,6].

## 2. Changing Approaches for Targeting Nutrition in CVD: Still Room for Improvement

The relevant role of nutrition for disease prevention and treatment was already understood in 1747, when James Lind, a Scottish surgeon in the Royal Navy, demonstrated the beneficial effects of citrus fruit for the treatment of scurvy in one of the first clinical trials. The first evidence that nutrition influences the onset and the progression of CVD came in 1908 from the Russian scientist Alexander Ingatowski, who demonstrated that high cholesterol intake caused the development of atherosclerosis in rabbits [7]. Since then, many studies were published that confirmed the role of a fat-enriched diet in the pathogenesis of atherosclerosis, leading to the formulation of the *cholesterol hypothesis* [8]. Following these observations, the first ecologic studies began to develop, such as the pioneering Seven Countries Study, which provided further insights on the impact of different lipids’ intake on CVD [9,10]. Moreover, a potential protective role of ω-3 polyunsaturated fatty acids (PUFA) also emerged from observational studies in Eskimos, among which CVD is a rarity [11]. Recently, it was also demonstrated that *n*-3 PUFA exert beneficial effects on endothelial progenitor cell biology [12].

Consequently, in 1957, when the American Heart Association (AHA) Nutrition Committee released the first dietary recommendations, they recognized that “diet may play an important role in the pathogenesis of atherosclerosis and the fat content and the total calories in the diet are probably important factors” [13]. This constituted a milestone of the nutrient-based approach for the prevention and treatment of CVD.

Despite its proven efficacy, this single-nutrient-based strategy appears not to be enough to contrast the onset and the progression of CVD. Indeed, there is growing evidence that, with few exceptions (ω-3 PUFA, sodium, *trans*-saturated fatty acids), single nutrients have effects of limited magnitude on chronic disease, compared with whole foods, or with complex integrated dietary interventions [14]. These and other considerations, such as the difficulties of translating single nutrient-based recommendations into an effective population-wide intervention, led to the advent of a different approach to address nutrition to reduce the burden of CVD, based on foods and dietary patterns rather than on single specific nutrients [15]. These dietary interventions take advantage of the beneficial effects of each of their multiple nutrient components, combining them into healthful diets that achieve greater net effects compared with most single nutrient supplementations. The nutrients and foods act additively and synergistically in the context of each dietary “recipe”, though maximizing the magnitude of their final beneficial effects [16].

Despite decades of nutritional research, by 2013 dietary-associated risk was still responsible for 37% of deaths and 24% of disability-adjusted life years (DALYs) for all ages and both sexes [3]. Notably, 9 out of 25 leading global risk factors for DALYs in 2013 were related to inappropriate eating habits (*i.e*., alcohol use, low intake of fruit, whole grains, vegetables, nuts and seeds, omega-3, fiber, excessive intake of sodium, and iron deficiency) [3]. Effective nutritional interventions, along with promotion of smoke discontinuation and regular practice of aerobic physical activity, are warranted as crucial elements of CVD prevention and regression. 

## 3. Dietary Patterns in Cardiovascular Disease

Despite the extraordinary progress in the treatment of CVD, our knowledge about the cardiovascular effects of diet is still regrettably limited. However, from the 1990s, with the transition from a nutrient-based to a dietary-based approach for addressing nutritional interventions in CVD, new promising data emerged from well-designed randomized trials and meta-analyses. Although the dietary recommendations endorsed by the major Cardiovascular Societies regarding the most widespread CVD, namely CAD and HF, are still based on little firm evidence, unprecedented progress was made in the last few decades in finding novel effective nutritional strategies for CVD prevention and treatment.

### 3.1. The Mediterranean Diet

In 1970, the American biologist Ancel Keys published the preliminary results of the Seven Countries Study, showing that populations dwelling on the shores of the Mediterranean Sea, in Greece, southern Italy, and the former Yugoslavia, had lower incidence of CAD and CVD in general [9]. Firstly described by Keys himself, the Mediterranean (MED) dietary pattern is rich in whole grains, fruit, vegetables, and low in meat, with a considerable amount of fat deriving from olive oil and nuts (Table 1) [17,18]. This diet seemed to be a possible determinant of the wide difference in CVD prevalence between Mediterranean populations and the Western cohorts in the Seven Countries Study.

#### 3.1.1. Mediterranean Diet and CAD Primary Prevention: From Observational Studies to the PREDIMED Trial

The first pilot studies began analyzing the association between adherence to MED diet and overall survival in the elderly population. In 1995, Trichopoulou and colleagues found out that adherence to MED diet, assessed through a food frequency questionnaire (FFQ) and summarized in a score (a MED score), was strongly associated with overall survival in 187 elderly Greeks. One point increase in the MED score was associated with a 17% increase in overall survival (*p* = 0.04) [20]. This finding was then confirmed in other three prospective cohorts from different geographical regions [21,22,23]. In 2003, Trichopoulou and colleagues published the results of probably the most important study on MED diet in the primary prevention of CAD and CVD. In a large population-based prospective study involving 22,043 Greeks enrolled in the European Prospective Investigation into Cancer and nutrition (EPIC), with a median follow-up of 44 months, a higher adherence to the MED diet was associated with an increased overall survival. Indeed, a 25% rise in the survival rate was observed every 2 points increase in the MED score assessed at baseline (hazard ratio (HR) 0.75, 95%, confidence interval (CI) 0.64–0.87; *p* < 0.001). The association with the MED score appeared to be evident for mortality from CAD (HR 0.64, 95% CI 0.47–0.94) and, although to a smaller extent, for mortality from cancer (HR 0.76, 95% CI 0.59–0.98), after adjustment for confounding factors [16]. Interestingly, no association emerged between mortality and each of the foods considered in the MED score, thus indicating that the total effect of the whole dietary regimen was stronger than any of the effects of its individual food components [16]. The contribution of the individual components of the MED diet to the overall effect were analyzed by Trichopoulou and her research group after an 8-year follow-up of the Greek cohort of the EPIC. The main contributors to the association of the MED score with mortality were moderate ethanol consumption, low consumption of meat products, high vegetable consumption, and high fruit and nut consumption (Table 2) [24].

These intriguing results were then replicated in larger cohorts worldwide: in 2339 European elderly adults from the Healthy Ageing: a Longitudinal study in Europe (HALE) population [25], in a group of 330,296 US residents enrolled in the National Institutes of Health (NIH)—American Association of Retired Persons (AARP) Diet and Health Study and in a cohort of 74,886 female nurses from the Nurses’ Health Study, all of which showed a strong association between adherence to a MED diet and lower all-cause and cause specific (CAD, stroke, CVD or cancer) mortality [26,27]. Noteworthy, the aforementioned studies were epidemiologic prospective studies, comparing cardiovascular outcomes and adherence to the MED diet, assessed through FFQs and expressed as scores. Thus, due to the lack of data from large randomized intervention trials, the evidence supporting the MED diet for the primary prevention of CAD was not enough solid to deserve a strong recommendation by the major Cardiovascular Guidelines. The AHA Guidelines on Lifestyle Management to Reduce Cardiovascular Risk published in 2013 consider the advice to eat a MED diet although judging the level of the supporting evidence as “Low” [19]. Most significantly, the 2012 European Society of Cardiology (ESC) Guidelines on Cardiovascular Disease Prevention recommend following a “healthy diet” rich in fruit, vegetables and fish, but do not mention the MED diet at all [28] (see Table 3 for a summary of the main dietary recommendations for CVD prevention).

Finally, in 2013 stronger evidence supporting the MED diet for the primary prevention of CAD came from the PREvenciόn con DIetaMEDiterránea (PREDIMED) trial. From 2003 to 2006, 7447 Spanish adults with high cardiovascular risk but with no diagnosis of CVD, were randomly assigned in a 1:1:1 ratio to one of three studied diets: a MED diet supplemented with extra-virgin olive oil, a MED diet supplemented with mixed nuts, or a control diet (advice to reduce dietary fat) [29]. Adherence was promoted through quarterly educational sessions and provision of extra-virgin olive oil or mixed nuts, and ensured by regular assessments of self-reported food intake and biomarker analyses. The primary endpoint was the rate of major cardiovascular events (*i.e*., myocardial infarction, stroke, or cardiovascular death). After a median follow-up of 4.8 years, the primary endpoint occurred in 96 subjects assigned to the MED diet with olive oil (adjusted HR 0.70, 95% CI 0.53–0.91, *p* = 0.009) and 83 subjects assigned to the MED diet with nuts (adjusted HR 0.70, 95% CI 0.53–0.94, *p* = 0.02), *versus* 109 in the control group. Stroke was the most significantly reduced event with the MED diet, followed by myocardial infarction (MI) [29]. Total mortality showed a non-significant trend towards reduction in the MED diet groups, compared with the control group [29]. Although with limitation regarding the possibilities of generalizing its results to non-Mediterranean populations, the 30% reduction of cardiovascular events seen with the MED diet in the PREDIMED trial is truly remarkable and strengthens the evidence in favor of recommending the MED diet for the primary prevention of CAD. Lately, some arguments have been raised about the possible role of lipid intake, from rapeseed oil margarine, olive oil or mixed nuts, in determining the benefits observed in the trials carried out on the MED diet for primary and secondary prevention of CAD [30]. Further randomized trials are needed to confirm or rule out this possibility.

#### 3.1.2. Mediterranean Diet and CAD Secondary Prevention: From Lyon Heart Study to Present Days

The Lyon Heart Study was a landmark study of the MED diet tested for the secondary prevention of CAD [31,32]. From 1988 to 1992, 605 survivors after a first MI were enrolled and randomized either to a control group, receiving dietary advice for a “prudent” low-fat diet, or the experimental group, undergoing an hour-long educational session about the MED diet and supplied a rapeseed oil based margarine comparable in composition to olive oil, but more palatable to the study population. Moderate alcohol consumption was allowed. Although serum lipids, body mass index (BMI) and blood pressure (BP) remained similar in the two groups, after a mean follow up of 27 months only three cardiac deaths occurred in the experimental group *versus* 16 in the control group (relative risk (RR) 0.27, 95% CI 0.12–0.59, *p* = 0.001), and overall mortality was eight subjects in the experimental group *versus* 20 in the control group (RR 0.30, 95% CI 0.11–0.82, *p* = 0.02) [31]. By the end of the study, after a mean follow-up of 46 months all the composite outcomes, combining cardiac death and non-fatal MI with other events, were significantly reduced in the MED group [32]. Although no other randomized trial was carried out for secondary prevention of CAD with the MED diet, prospective studies published in the last 15 years confirmed the findings of the Lyon Heart Study [33,34]. Intriguingly, one randomized trial comparing dietary intervention (101 patients, 50 randomized to a low-fat diet and 51 to the MED diet) *versus* usual post-MI care, found that dietary intervention *per se* was beneficial after MI [35]. The relatively short time spent for dietary advice and the lenient follow-up schedule in the Lyon Heart Study suggest that benefits from dietary interventions on CVD are achievable with limited effort and are feasible on large-scale. Secondly, dietary intervention showed a complementary beneficial role beside pharmacological treatment in post-MI care. Currently, the MED diet appears to be the only dietary pattern supported by a large randomized trial for the secondary prevention in patients with established CAD [36,37] (See Table 4 for a summary of the main dietary recommendations for CAD secondary prevention).

#### 3.1.3. Mediterranean Diet and Heart Failure

Heart failure is a CVD characterized by a severe prognosis. Moreover, patients with heart failure often have comorbidities that negatively affect the prognosis, including kidney disease, anemia, respiratory disorders and depression [38,39,40]. Chronic heart failure progression is modifiable using therapies that antagonize adverse neuro-hormonal pathways (beta-blockers, angiotensin-converting-enzyme inhibitors, angiotensin receptor blockers, and mineralocorticoid antagonists) while diuretics are effective in treating congestion and HF symptoms [41,42]. Despite the current improvement in the management of chronic heart failure, none of the available treatments has demonstrated to improve in prognosis in acute heart failure, except a new vasodilator whose trial is still underway [43,44]*.*

Beside the above mentioned therapies, there is actually a solid rationale for the beneficial effects of nutritional interventions on HF, especially regarding the MED diet (see Table 5 for a summary of the main dietary recommendations for HF) [45,46].

Notably, multiple risk factors (e.g., hypertension, diabetes) as well as pathophysiological mechanisms (e.g., systemic inflammation, neurohormonal activation) may be positively influenced by the MED diet [47,48].

A meta-analysis by Nordmann and colleagues, showed significant benefits of the MED diet, compared with low-fat diets, in reducing BMI, systolic blood pressure (SBP), fasting plasma glucose, total cholesterol, and high-sensitivity C-reactive protein [49]. Moreover, MED diet adherence was associated with lower serum concentrations of biomarkers related to inflammation and endothelial dysfunction, in a cohort from the Nurses’ Health Study [50], and with lower serum lipids and oxidized LDL, in a randomized sample from the PREDIMED trial [51]. It is therefore in line with these findings, that data collected from prospective cohorts showed an association between adherence to the MED diet and lower incidence of HF both in men (multivariable RR for the highest *vs.* lowest quartile of MED score 0.69, 95% CI 0.57–0.83) and in women (RR 0.79, 95% CI 0.68–0.93, *p =* 0.004) [52,53]. 

Given these results, it is therefore not surprising that MED diet was shown to influence also HF progression and mortality. An interesting association of the MED diet with an improvement of ventricular function was suggested by a study conducted by Chrysohoou and colleagues, demonstrating that high adherence to the MED diet was associated with improvement in left ventricular ejection fraction (LVEF) and diastolic function [54,55]. Additionally, data from the PREDIMED study showed a reduction in serum biomarkers related with HF in the groups randomized to the MED diet (mean NT-proBNP reduction: −84.7 pg/mL, 95%CI −145 to −24.5, *p* = 0.006) [56]. Other prospective studies provided insights on the favorable association of MED diet with lower incidence of sudden cardiac death (SCD), one of the main causes of death in patients with HF. In a study by Bertoia *et al.* on 93,000 women enrolled in the Women’s Health Initiative, MED diet, but not DASH diet adherence, was associated with lower risk of SCD (highest to lowest quintile HR 0.64, 95% CI 0.43–0.94) [57], a result emphasized also in a cohort from the Nurses’ Health Study [58]. Finally, a considerable amount of studies showed interesting associations of MED diet adherence with lower incidence of various conditions that are usually present as comorbid diseases in HF patients [59]. The results of multiple prospective trials conducted until 2013 are well summarized in a meta-analysis by Sofi and colleagues showing substantial benefit of the MED diet on overall, cardiovascular and non-cardiovascular mortality [60]. A consistent number of studies, published during the past decade, further confirmed the association of MED diet adherence and favorable outcome of various HF comorbidities, like diabetes, metabolic disease and obstructive lung disease [61,62]. 

All those data may explain the reduced risk of death observed in HF patients following a MED diet pattern. This reduced risk of death was demonstrated in a population of 37,308 men from the Cohort of Swedish Men (RR of HF mortality: 0.55; 95% CI 0.31–0.98) [52], and observed as a non-significant trend among 3215 female participants of the Women’s Health Initiative (RR highest to lowest quartile: 0.85; 95% CI, 0.70–1.02) [63]. Although there is need of more conclusive data from randomized intervention trials, these results provide encouraging evidence on the benefits of a MED-style diet for HF patients.

The pathophysiological mechanisms that may explain these beneficial effects of diet in HF are not limited to those previously mentioned. In addition, several micronutrient deficiencies (*i.e.*, iron, coenzyme Q10, vitamin D, thiamine, and amino acids) have been described in HF [64,65] that may benefit from dietary supplementation, although specific studies led to controversial results [64,66,67]. Likely, an integrated dietary intervention, with a well-balanced food composition along with a comprehensive micronutrient content could be the most successful nutritional strategy in HF.

#### 3.1.4. A Cluster of Definitions: What Does *Mediterranean Diet* Mean Today?

Given the social and cultural changes in alimentary habits and tastes from 1960s to present days, the current MED diet is not the same as the one that Cretan people ate at the time of the Seven Countries Study. As a consequence various studies, carried out in different decades, used different FFQ and MED scores. A brief description of the current MED diet based on the most significant studies and trials that demonstrated its properties has been provided by the AHA in the Guidelines on Lifestyle Management to Reduce Cardiovascular Risk and is summarized in Table 1 [19]. Recently, a new MED diet pyramid based on scientific evidence and epidemiological studies was elaborated to summarize the MED dietary pattern as it can be applied to present days and adapted to different geographical, cultural and socio-economic contexts [68].

### 3.2. The DASH Diet

In the 1990s, the prevalence of hypertension, one of the main determinants of CVD, had already reached the proportions of an epidemic among the American population [69]. Following the observation that vegetarians tended to have lower BP values than non-vegetarians [70], a Collaborative Research Group led by Lawrence Appel tested the effects of a diet rich in fruit, vegetables and low-fat dairy foods on blood pressure in a multicenter randomized feeding study: the Dietary Approach to Stop Hypertension (DASH) trial. This trial enrolled 459 adults with SBP lower than 160 mmHg and diastolic blood pressure (DBP) of 80 to 95 mmHg, not on BP-lowering medications. Participants were randomly assigned to eight weeks feeding with a control diet, similar in composition to the average American diet, or a diet rich in fruit and vegetables, or a “combination” diet (hereafter referred to as the DASH diet) rich in fruit, vegetables, and low-fat dairy products with a reduced content of saturated and total fat (components of the DASH diet are listed in Table 1). After 8 weeks, compared with the control diet, the fruits-and-vegetables diet reduced SBP by 2.8 mmHg (*p* < 0.001) and DBP by 1.1 mmHg (*p* = 0.007), and the combination diet reduced SBP by 5.5 mmHg and DBP by 3 mmHg (*p* < 0.001 each) [71]. The effects of the combination diet were even more pronounced among the 133 subjects with hypertension, which experienced a mean reduction in SBP of 11.4 mmHg and in DBP of 5.5 mmHg compared with the controls (*p* < 0.001 each) [71]. The DASH trial presented a strong study design leading to minimization of potential biases. A significant strength of the study was the fact that all meals were prepared in the research kitchen, enabling full control of the food and nutrient composition of the studied diets.

Further analyses showed that the DASH diet reduced total (−13.7 mg/dL) and LDL (−10.7 mg/dL) cholesterol (all *p* < 0.0001) [72]. A subgroup analysis demonstrated a greater effect on SBP in African Americans (−6.8 mmHg) than in whites (−3.0 mmHg) (*p* < 0.05) [73]. In addition, data from one-year follow-up of the study population, showed sustained reductions of BP and a positive influence over time on eating habits in DASH diet group [74]. Subsequent studies proved that the DASH diet does not exert a simple “cosmetic” effect on BP values, but it is also contrasts inflammation and the detrimental effects of hypertension on organ damage. In 2009, Jacobs and colleagues showed that the DASH dietary pattern led to a reduction of albumin excretion rate (AER) [75]. This finding was confirmed in the CARDIA study, that demonstrated an association between scarce adherence to the DASH dietary pattern and obesity with incident microalbuminuria in a young healthy population [76].

#### 3.2.1. Unity Makes Strength: The DASH-Sodium Trial

The DASH-sodium trial was designed to assess whether a low-sodium content could improve the benefits of the DASH diet alone [77]. Participants were assigned to a diet, either DASH or control, each combined with high (*i.e*., 150 mmol/day of sodium, reflecting typical consumption in the US), or intermediate (*i.e*., 100 mmol/day, corresponding to the upper limit of the National Recommendations in 1997), or low sodium content (*i.e*., 50 mmol/day, a level hypothesized to produce an additional lowering in BP). In this trial, the reduction of sodium intake produced an additional significant BP lowering effect both combined with the DASH diet (3 mmHg from high to low sodium level, *p* < 0.01), or the control diet (6.7 mmHg from high to low sodium level, *p* < 0.001). The BP lowering effect of reduced sodium intake was observed in all the analyzed subgroups, though more pronounced among black, hypertensive and female subjects [78].

#### 3.2.2. Theme and Variations: The OMNI-Heart Trial

After the encouraging results of the previous DASH trials, the Optimal Macro-Nutrient Intake Heart trial (OMNI-Heart) was carried out to test the potential benefits of a DASH diet with varying content in macronutrients, on CVD risk [79]. A sample of 164 pre-hypertensive or mild hypertensive subjects with LDL cholesterol (LDL-C) <220 mg/dL, triglycerides (TG) <750 mg/dL, not on medications influencing BP or blood lipids, was randomized to a sequence of three diets. One of the diets was a carbohydrate-rich diet similar to the DASH diet, the other two were modified DASH diets: a protein-rich diet, and an unsaturated fatty acids-rich diet (see Table 1). All three dietary patterns were produced significant lowering of BP and LDL-C values from baseline, with greater results with the protein- and unsaturated fatty acids-rich diets [80].

#### 3.2.3. DASH Diet from Risk Factors Reduction to CAD Prevention

The aforementioned DASH diet trials showed remarkable effects on BP and lipid profile that encompass all degrees of hypertension, making it a useful tool for population-wide interventions aimed at reducing cardiovascular risk. Notably, the effects of the DASH diet were more pronounced among hypertensive subjects compared with the normotensive, thus making it an even more appropriate strategy for initial treatment of hypertension [71]. Moreover, subsequent studies demonstrated that the DASH diet led to further BP decrease compared with the pharmacological therapy alone, when added to either losartan or candesartan [81,82], thus extending its benefits also in patients on BP-lowering drugs. Finally, the more pronounced effects of the DASH diet among black patients, make it an useful tool to reduce cardiovascular risk in developing countries, whose results have already been encouraging in some pilot studies [83].

Besides risk factor reduction, the DASH diet has also other potential benefits in the setting of CAD prevention. In a prospective cohort of 88,517 females from the Nurses’ Health Study, Fung and colleagues found that a high DASH adherence score was associated with less inflammation (assessed as C-reactive protein and interleukin-6 serum concentration) [84]. Interestingly, in a cross-sectional study on 148 adults undergoing coronary angiography, greater adherence to the DASH diet was associated with lower concentrations of asymmetrical dimethyl-arginine, a marker of endothelial dysfunction, which was associated with the presence of CAD [85]. Moreover, in 2008, Fung and colleagues demonstrated that adherence to a DASH diet, assessed seven times during 24 years of follow-up in a prospective cohort of 88,517 female nurses without prior history of CVD, was associated with significantly lower risk of CAD (RR across quintiles 1.0, 0.99, 0.86, 0.87, and 0.76, *p* < 0.001 for trend) and stroke (RR across quintiles 1.0, 0.92, 0.91, 0.89, and 0.82; *p* = 0.002 for trend) [84]. Thus, given the strength of the DASH trial findings, supported also by subsequent studies showing their successfully reproducibility in clinical practice [86], in 2013 the AHA Guidelines on lifestyle management to reduce cardiovascular risk recommended the DASH diet with “strong” level of evidence (LOE) to reduce cardiovascular risk [19]. Most importantly, the DASH diet was also associated with increased survival. In 2009, Parikh and colleagues found an association with DASH diet adherence and lower all-cause mortality among 5,532 hypertensive adults in the Third National Health and Nutrition Examination Survey (HR 0.69, 95% CI 0.52–0.92; *p* = 0.01) [87].

The above mentioned studies confirm the beneficial effects of this favorable dietary pattern not only on BP values, but also on inflammation and on the micro- and macrovascular damage, multiple additive beneficial effects that efficiently sum up to achieve the final goal of reducing cardiovascular events. Although randomized trials are needed to establish whether the DASH diet could be beneficial in the primary and secondary prevention of CAD there is a strong rationale supporting its potential beneficial effects also in that context (see Table 3 and Table 4 for a summary of the main dietary recommendations for CAD prevention).

#### 3.2.4. DASH Diet and Heart Failure

The DASH diet exerts positive effects also in patients with HF. Recently, Levitan and colleagues demonstrated that high adherence to a DASH diet was associated with lower incidence of HF, compared with low DASH diet adherence, in a prospective cohort of women from the Swedish Mammography Cohort (RR highest to lowest quartile 0.63, 95% CI 0.48–0.81; *p* for trend < 0.001) [88], and with lower HF deaths and hospitalizations in a prospective cohort of men from the Cohort of Swedish Men (22% lower rate of HF events in the highest *vs.* lowest quartile, 95% CI 5%–35%, *p* for trend = 0.006) [89]. These results have been confirmed by a subsequent meta-analysis displaying that following the DASH diet can significantly protect against the most widespread CVD, reducing the risk of CVD, CHD, stroke, and HF by 20%, 21%, 19% and 29%, respectively (all *p <* 0.001) [90]. Finally, an analysis by Levitan *et al.* in a prospective cohort from the Women’s Health Study observed a relative risk reduction of HF mortality of 16% across quartiles of the DASH diet score (RR of HF mortality 0.84, 95% CI 0.70–1.00; *p* for trend = 0.01) [63].

In the past decade, some small studies have explored the mechanisms leading to this reduction in HF incidence, progression and mortality. First, as previously discussed, hypertension is one of the main contributors to the pathogenesis of HF, especially HF with preserved ejection fraction (HFpEF) [91]. In a small study of 13 patients with hypertension and stable HFpEF, a sodium restricted DASH diet resulted in significant reduction of BP, along with a reduction in carotid-femoral pulse wave velocity, an index of arterial stiffness [92]. A DASH dietary pattern was also associated with improvements in left ventricular diastolic function, arterial elastance, and ventricular-arterial coupling in patients with HFpEF [93]. Finally, using targeted metabolomics to explore metabolite changes, proof was provided that a sodium restricted DASH could improve myocardial energy substrate utilization in patients with HFpEF [94]. The DASH diet was also demonstrated to be positively associated with left ventricular contractile function, thus providing potential benefits to patients suffering of HF with reduced ejection fraction (HFrEF). In the Multi-Ethnic Study of Atherosclerosis (MESA), a 1-unit increase in DASH diet score was significantly associated with an increase in stroke volume (+0.10 mL/m^2^), with a non-significant trend towards an increase in left ventricular ejection fraction (+0.04%, *p* = 0.08) [95]. In addition, the DASH diet improved symptoms and quality of life (QoL) in patients with established symptomatic HF. In a small randomized trial of 48 stage C chronic HF patients, an improvement in exercise capacity (292 m *vs*. 197 m; *p* = 0.018) and QoL scores (21 *vs.* 39; *p* = 0.006) were observed in patients randomized to the DASH diet [96]. Finally, early studies provided insights about a possible advantageous effect of this diet in relieving congestion often associated with HF. In 2003 Akita *et al.* performed an analysis of the BP-natriuresis relationship in patients enrolled in the DASH-sodium trial. The results showed that the DASH diet had the effect of steepening the x-y relationship between BP and natriuresis (slope was increased from 29.5 ± 3.4 to 64.9 ± 13.1 mmol/day/mmHg, *p* = 0.0002), providing the first evidence of a possible natriuretic effect of the DASH diet [97].

After the publication of these encouraging data, randomized trials are needed to get more conclusive data on the beneficial effects of the DASH diet in patients with preclinical or established HF, thus enabling the formulation of more specific guidelines to deal with the complex problem of nutrition in patients with HF (see Table 5 for a summary of the main dietary recommendations for HF).

### 3.3. The Next Future: Promising Dietary Patterns

Beside the above-mentioned dietary patterns, some other approaches have been described and tested that may represent valid tools for CVD prevention and treatment. They are either empirically derived dietary patterns, or hypothesis driven dietary patterns [5]. The former are dietary patterns observed to be beneficial on CVD in epidemiological studies, which have been subsequently analyzed and then tested in prospective or interventional studies. The latter are either based on diet quality or on adherence to dietary guidelines or are groups of food expected to act synergistically on a common target, and thus are artificial dietary models. In the next future, these diets will be possibly tested on hard endpoints in large randomized trials, hopefully confirming the encouraging results of the early studies.

#### 3.3.1. Empirically Derived Dietary Patterns

**The Japanese Diet.** Following the observation that Japanese inhabitants of the Okinawa Prefecture have the longest life expectancy in Japan and likely in the world [98], it has been hypothesized that their traditional diet rich in fish, seaweed, soybean products, vegetables and green tea, may convey health benefits. Although the single components of this diet have been associated with cardiovascular benefits [99,100,101] only few studies investigated the effects of the whole Japanese dietary pattern on CVD. Interestingly, despite being associated with higher prevalence of hypertension, probably due to its high sodium intake, the traditional Japanese dietary pattern, after adjustment for potential confounders, showed to reduce the risk of CVD mortality [102]. On the other hand, a recent study by Niu *et al.* showed that a traditional Japanese diet was associated with lower BP, although the sodium content of the diet pattern followed by the study participants was not specified [103]. Taken together, these results show that the Japanese dietary patter seem to exert favorable effects on CVD regardless of its effects on BP. These preliminary results deserve further research to better characterize the benefits of this dietary pattern while providing new insights on the role of BP on CVD.

**The Nordic Diet.** During the last five years, the effects of a Nordic Diet (ND) including oily fish (salmon and mackerel), vegetables, roots, legumes, fruits, berries and wholegrain cereals (oat, rye, and barley) [104], were studied in epidemiological studies and randomized trials. The first randomized trial on ND, carried out in 2007–2008, showed a significant reduction in total cholesterol (−0.98 ± 0.75 mmol/L, *p <* 0.0001) and LDL-C (−0.83 ± 0.67 mmol/L, *p <* 0.001) as well as in weight (−3 ± 1.86 Kg, *p <* 0.001) and SBP (−6.55 ± 13.18 mmHg, *p* = 0.008) in six weeks, among those randomized to ND compared with controls [105]. Following this trial, other feeding trials confirmed the effects of the ND on hypertension and blood lipids, and showed that ND exerts positive effects also on inflammation, insulin sensitivity and body weight [106,107]. Adherence to the ND, assessed with FFQ, was even associated with lower risk of all-cause mortality in two large cohort studies [108,109]. However, a recent cohort study on a large sample of Swedish women did not find a significant association between adherence to a ND and a reduction of risk of CVD [110]. Even though a lack of accuracy in the ND adherence score has been proposed to explain this surprising result [111], new prospective studies and trials are warranted to clarify the effects of this dietary pattern.

**The Vegetarian Diet**. The association between a vegetarian diet and lower BP values has been known since the 1970s, and was actually confirmed by the lower BP values observed in the vegetable-rich-diet arm of the DASH trial [70,71]. A recent meta-analysis observed that a vegetarian diet significantly lowered blood cholesterol levels, LDL-C, HDL-C, and non-HDL-C, without affecting TG [112]. In another meta-analysis, vegetarian diet reduced significantly the risk of incidence and/or mortality from ischemic heart disease (RR 0.75; 95% CI, 0.68–0.82) [113]. However, other single studies and pooled analysis failed to confirm these results [114,115]. Similar to the Japanese diet, these results underscore the concept that BP cannot be linked *a priori* to cardiovascular outcomes, and that further prospective and randomized trials are needed.

#### 3.3.2. Hypothesis Driven Dietary Patterns

**The Portfolio Diet.** The “portfolio” diet is a dietary approach meant to achieve effective cholesterol reduction through a combination diet of functional foods or foods containing specific therapeutic components. The basic idea, first proposed in 1999, was to combine into one diet viscous fiber, soy, almonds, plant sterols and stanols [116]. Various studies tested different food combinations obtaining remarkable reductions in LDL-C ranging from 4% to 35% [117]. Noteworthy, in a randomized study, Jenkins and colleagues assigned 48 subjects in a 1:1:1 ratio to a diet very low in saturated fat (control), or the same diet plus lovastatin 20 mg (statin), or the portfolio diet. After one month, the control, statin, and dietary portfolio groups showed mean decreases in LDL-C of 8.0% ± 2.1% (*p* = 0.002), 30.9% ± 3.6% (*p <* 0.001), and 28.6% ± 3.2% (*p <* 0.001), respectively, thus demonstrating that the efficacy of this dietary portfolio was comparable to a statin therapy [118]. Further studies demonstrated that dietary portfolio reached a significant BP-lowering effect, comparable to that of the DASH diet [119], and a positive effective risk factor management in patients with established CAD [120].

**The Glycemic Index Diet.** In 1981, Jenkins and colleagues first published a paper on the effects of various food on blood glucose levels [121]. The authors concluded that a diet based on GI might be particularly promising for nutrition in diabetics or in patients with a metabolic disease [121]. During the following years a dietary approach based on GI or glycemic load (GL, *i.e*., the GI of a food multiplied by its carbohydrate content) was tested on various conditions, and also in CVD. Some meta-analysis reported that GI was associated with significant increased risk of CVD [122,123], but other studies led to conflicting results [124,125]. Interestingly, a six-month randomized controlled trial conducted in 122 overweight and obese adults (the GLYNDIET study), showed that a low-GI and energy-restricted diet may be more effective than a high-GI and low-fat diet at reducing body weight and controlling glucose and insulin metabolism [126]. These results suggest that a GI-based diet may be more useful in diabetic patients than in non-diabetics. Further studies are needed to clarify the effects of GI-based diets on CVD according to gender, weight and concomitant metabolic diseases.

## 4. Beyond Nutrients: Which Is the Optimal Amount of Salt to Flavor a Healthful Diet?

Current guidelines recommend to reduce sodium intake either for primary or secondary prevention of CAD and CVD (Table 3 and Table 4). The recommended targets vary from 4 to 5 g of salt per day, corresponding to 1550–2000 mg of sodium. When considering HF, some guidelines even recommend salt restriction to 1–2 g per day, in case of advanced symptoms [127]. In fact, the DASH-sodium trial [78] and large randomized trials, such as the INTERSALT and INTERMAP [128,129], provided evidence of the beneficial effects of a low sodium diet for hypertension, but should sodium restriction be recommended also to non-hypertensive patients? While some authors have claimed that a salt-restricted diet could reduce cardiovascular risk [130,131], others trials and meta-analyses supported the opposite viewpoint [132,133].

In 2013, the National Institute of Medicine reviewed the existing evidence for sodium restriction in CVD and concluded that there was not sufficient evidence from solid studies to support the recommendation of sodium restriction to prevent and treat CVD, except from hypertension. Actually recent studies in HF setting provided evidence that sodium restriction may even worsen clinical outcomes. In a prospective study of 244 patients with HF, Song and colleagues demonstrated that patients in NYHA class I/II with <2 g/day sodium intake had a 3.7-times higher risk (*p* = 0.025) for hospitalization or death than those with 2–3 g/day sodium intake after controlling for covariates. Conversely, in NYHA class III/IV, >3 g/day sodium intake predicted shorter event-free survival (*p* = 0.044), whereas there was no difference in survival curves between patients with <2 g/day and those with 2–3 g/day sodium intake [134]. Recently, a study by Doukky *et al.* has been published that suggests that sodium restriction may increase hospitalizations in patients with NYHA II/III HF, especially in patients not receiving angiotensin-converting enzyme inhibitor or angiotensin receptor blocker (HR: 5.78; 95% CI: 1.93 to 17.27; *p* = 0.002), thus suggesting that sodium intake produces not only hemodynamic but also neurohormonal changes [135].

The publication of the document by the National Institute of Medicine and of the subsequent studies, gave the beginning to a new wave of research on potential benefits and drawbacks of sodium intake in CVD. Some randomized trials are currently ongoing to determine the actual effects of salt in different cardiovascular diseases [136].

## 5. Dietary Interventions and the Real World: The Complex Issue of Translating Knowledge into Practice

Since their publication, the dietary patterns supported by the strongest evidence, namely the MED and the DASH, have been recommended by most Cardiovascular Societies worldwide. However, the adherence of general population to these dietary patterns is very low and with a temporal trend towards divergence from these diet models, especially in the subgroups expected to receive greater benefits from these diets, thus neutralizing the potential benefits of these “weapons” [137].

There are several reasons that may explain poor adherence to a virtuous dietary pattern. In first place, despite health promotion programs, secular trends and food industry induce an increase in consumption of highly-refined energy-dense products instead of low-fat, fresh food [138]. Secondly, recommended diet patterns are, at various extents, more costly than the average Western food patterns [139,140,141]. For instance, DASH diet costs $130 per week for a family of four, having been classified in a “low“ to “moderate” cost category according to the USDA estimates [73]. Notably, a recent study of 2181 Spanish subjects, found out that every 1€ increase of the diet cost per 8.36 MJ was associated with an average 300 hg decrease in body weight and a 0.1 Kg/m^2^ decrease in BMI (*p* = 0.02 and *p* = 0.04, respectively), thus confirming that improvements in diet quality entail increases in diet costs [142]. Thirdly, fresh produce and groceries appear to be less available in urban communities, where most of the high-risk population dwells [143]. In fourth place, the lack of palatability of this kind of diet has been proved a further reason of scarce adherence to a healthful kind of diet, especially among African Americans [144]. Finally, education, social status and the presence of other lifestyle- and behavior-related cardiovascular risk factors have been associated with poor adherence to healthful dietary pattern. A recent analysis of the PREDIMED trial cohort at baseline, showed that little education, a larger waist-to-height ratio, diabetes, low physical activity, single, divorced or separated social status, and current smoking were associated with lower adherence to a MED diet [145]. Actually adherence could be one of the main barriers to the beneficial effect of the dietary approaches in CVD. A recent study by Wong and coll. showed that counseling towards choosing a DASH dietary pattern was not sufficient to obtain significant blood pressure reductions in a Chinese cohort of mild hypertensive patients, thus underscoring the importance of finding an efficient delivery model to get the expected benefits from an intervention [146]. In addition to the adherence issue, it is worth considering that potential benefits of some food categories may be outweighed by emerging drawbacks, as in the case of oily fish consumption and the risk associated with its content in toxic lipophilic organic contaminants (e.g., organochlorins) and heavy metals (e.g., mercury) due to ocean pollution [147].

Awareness of the reasons of poor efficacy is mandatory for planning successful population interventions aimed at reducing the burden of CVD by targeting the main modifiable risk factor [3]. Potential interventions should not concentrate on single risk factors, but embrace a comprehensive approach and broad recommendations, to maximize results in large populations, as many studies and trials point out [148,149]. Knowledge of effective dietary patterns and recommendations should not be considered a finishing point: finding correct strategies to effectively deliver evidence-based dietary recommendations to the population should be considered as important as dietary knowledge. Integration of dietary approaches with effective health policies will provide a low-cost support to high-cost drugs and device strategies in helping to improve cardiovascular outcomes worldwide.

## 6. From Populations to Individuals: Towards a Tailored Diet Approach

Clinical trials and prospective studies of dietary patterns in CVD showed the effects of different diets on heterogeneous populations. Subsequently, subgroup analyses demonstrated that the effects of a dietary pattern vary among different subgroups of people according to sex, age, race, and other individual factors. Moreover, the main individual pathology, the presence of comorbidities and of different risk factors contributes to the different individual responses to a diet regimen. Beside these determinants of the response to different nutrient combinations there is another one, which is receiving growing attention: the individual genetic profile.

Nutrigenetics, an emerging branch among nutritional sciences, analyzes the interaction of diet with common gene variants of candidate genes that determine different responses to dietary interventions [150]. Significantly, several genes have been identified whose variants, when combined with various dietary inputs, determine different susceptibility to various conditions like dyslipidemias and atherogenesis [151], activation of inflammatory pathways [152], or diabetes and metabolic disease [153].

Recently, data from large feeding trials like the DASH and PREDIMED trials were analyzed and compared with known genes affecting CVD, to clarify whether there are genetic determinants of the efficacy of these dietary patterns, and eventually to determine if there are genetic responders and non-responders to these diets. In 2013, Corella and colleagues published the results of an analysis of Transcription factor 7-like 2 (TCF7L2) polymorphisms among the participants of the PREDIMED trial [154]. The product of TCF7L2 is a high-mobility box-containing transcription factor that plays a role in activating multiple genes, and the rs7903146C polymorphism (more than the rs7903146T) is one of the most influencing genetic variants for type 2 diabetes risk [155]. Notably, when adherence to the MED diet was low, TT homozygotes had higher fasting glucose concentrations (132.3 ± 3.5 mg/dL) than CC and CT individuals (127.3 ± 3.2 mg/dL, *p =* 0.001), but when adherence was high, this increase was not observed (*p* = 0.605). This modulation was also observed for total cholesterol, LDL-C, and TG (*p* interaction < 0.05 for all). Moreover, compared with CC, TT subjects had a higher stroke incidence in the control group (adjusted HR 2.91, 95% CI 1.36–6.19, *p =* 0.006), and dietary intervention with MED diet reduced stroke incidence in TT individuals (adjusted HR 0.96, 95% CI 0.49–1.87, *p* = 0.892) [154]. This provided evidence that a dietary pattern, the MED diet, can overrule the metabolic and cardiovascular genetic risk associated with individuals carrying particular genetic polymorphisms. Later, Ortega-Azorìn and colleagues showed that individual genetic profile could also act synergistically with a dietary pattern in determining beneficial effects. In a sample from the PREDIMED trial they observed that a variant (rs3812316) in the MLXIPL gene encoding the carbohydrate response element binding protein and associated with lower serum TG, had cumulative beneficial effects when combined with high adherence to the MED diet [156]. Additionally, a dietary pattern was shown to influence the development of CVD also by inducing changes in the individual transcriptomic response of genes involved in cardiovascular risk. In a small subset of subjects from the PREDIMED trial, Castañer and colleagues performed an analysis of multiple genes’ expression profile and observed that MED diet either supplemented with olive oil or mixed nuts induced a variation in the transcriptomic response modulating 12 of the 18 signaling pathways analyzed [157]. The influenced pathways were involved in cardiac hypertrophy, renin-angiotensin-aldosterone system (RAAS), nitric oxide signaling, atherogenesis, and cardiac β-adrenergic signaling. After adjustment, 9 pathways resulted modulated by one or both variants of the MED diet and none of the pathways remained modulated by the low-fat control diet.

For these reasons, knowledge of the individual genetic risk may be useful to target appropriate specific dietary interventions to override genetic risk or to favorably change the individual gene expression profile. Data supporting this novel aspect of dietary interventions comes also from the DASH trial. Recently, Chen and colleagues demonstrated that the DASH diet was associated with an increase in plasma renin activity (PRA) among subjects enrolled in the DASH trial [158]. This acts as a counter regulatory mechanism that blunts the BP lowering effect of this dietary pattern. The role of beta-2 adrenergic receptors (β2-AR) mediated vasodilation in response to adrenergic agonists and renin secretion in the juxtaglomerular cells has been extensively studied in past years [159,160]. Recently, Sun and colleagues analyzed in the DASH study population the G46A (Gly16Arg) variant of β2-AR, which is associated with impaired agonist mediated receptor downregulation and desensitization, low PRA, and salt-sensitive hypertension. Homozygosis for the A allele was associated with greater SBP reduction when combined with high adherence to the DASH diet whereas GG homozygotes showed no significant SBP change due to an increased PRA and aldosterone concentrations [161]. This study provides further evidence of a possible future application of patient genotyping to tailor dietary interventions to fit individual needs.

**Figure 1:**
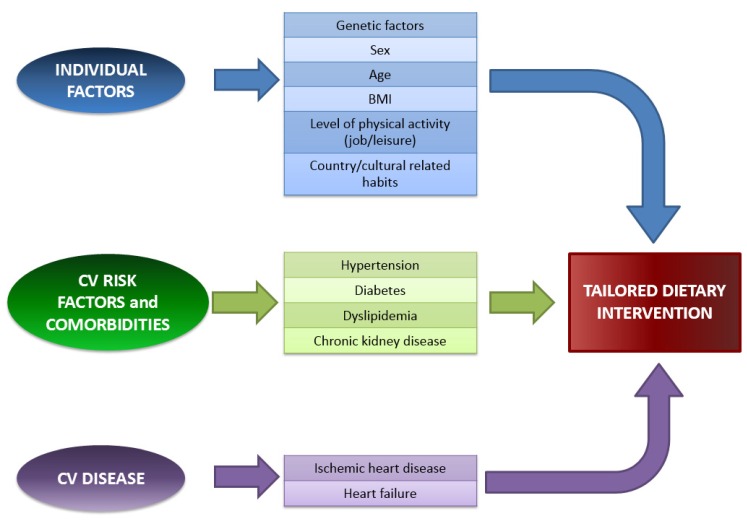
Factors to consider for tailoring dietary interventions in patients with CVD. BMI, Body Mass Index; CV(D), Cardiovascular (Disease).

To summarize, a one-size-fits-all nutritional intervention may be a limited approach in patients with CVD. Indeed dietary requirements differ not only between primary and secondary prevention of CAD or HF, but also between different individuals. In the future, a personalized and tailored intervention may reach greater benefits, taking into account also individual genome, beside social geographical and cultural factors, the presence of CV risk factors, comorbidities and special needs related to the specific CV disorder (Figure 1).

Further investigations and well-designed clinical trials are warranted in this field in order to ascertain the clinical efficacy, the impact on outcomes and the cost-effectiveness of this kind of interventions, that may broaden the fan of available tools for the clinician, so that is possible to better reach different at-risk populations.

## 7. Conclusions

A dietary approach to nutritional interventions in CVD had proved to be an effective strategy resulting in strong and tangible results. The aforementioned studies indicate that synergistic effects of food combined into a dietary pattern provide the maximum benefit obtainable from nutrition. In the next years, further randomized trials may increase our knowledge on the effects of different food combinations on hard cardiovascular outcomes. This will certainly help Cardiologists and General Physicians in prescribing a “tailored” dietary pattern to each patient, thus providing a complimentary therapy acting together with drugs and devices to improve health and survival.

## Figures and Tables

**Table 1:** MED diet and DASH diet composition [19].

MED Diet	DASH Diet
Although there is no uniform definition of the MED diet in randomized trials and cohort studies, the most common features of diets in these studies were the following: -High content in fruits (particularly fresh), vegetables (emphasizing root and green varieties), whole grains (cereals, breads, rice, or pasta), and fatty fish (rich in ω-3 PUFA); -Low content in red meat (emphasizing lean meats); -Substituted lower-fat or fat-free dairy products for higher-fat dairy foods; -Used oils (olive or canola), nuts (walnuts, almonds, or hazelnuts), or margarines blended with rapeseed or flaxseed oils in lieu of butter and other fats.	-High in vegetables, fruits, low-fat fermented dairy products, whole grains, poultry, fish, and nuts; -Low in sweets, sugar-sweetened beverages, and red meats; -Low in saturated fat, total fat, and cholesterol; -Rich in potassium, magnesium, and calcium; -Rich in protein and fiber. **DASH VARIATIONS** In the OMNI-Heart trial, 2 variations of the DASH dietary pattern were compared with DASH: -One that replaced 10% of total daily energy from carbohydrate with protein (mainly non-meat proteins); -Another that replaced the same amount of carbohydrate with unsaturated fat (manly from monounsaturated fatty acids).

DASH, Dietary Approach to Stop Hypertension; MED, Mediterranean, OMNI-Heart, Optimal Macro-Nutrient Intake Heart trial; PUFA, Polyunsaturated Fatty Acids.

**Table 2:** Components of the MED diet score and their contribution to the association between the MED score and overall mortality in the Greek cohort of the EPIC [21].

Dietary Components of MED Score	Influence on Survival
Ethanol intake (moderate)	24%
Meat and meat products intake (low)	17%
Vegetables intake (high)	16%
Fruits and nuts intake (high)	11%
Monounsaturated:saturated fat ratio (high)	10%
Legumes intake (high)	10%
Dairy products intake (low)	5%
Cereals intake (high)	5%
Fish and seafood (low)	n.s.

EPIC, European Prospective Investigation into Cancer and nutrition; MED, Mediterranean.

**Table 3:** ESC and ACC/AHA dietary recommendations for risk factor management and primary prevention of CVD.

Society	Diet Recommendations for CVD—Primary Prevention	COR/LOE
**European Society of Cardiology (2012), [28]**	A healthy diet is recommended as being the cornerstone of CVD prevention.	I B
	Energy intake should be limited to the amount of energy needed to maintain (or obtain) a healthy weight (BMI < 25 kg/m^2^).	-
	Saturated fatty acids to account for <10% of total energy intake, through replacement by PUFA.	-
	*Trans* unsaturated fatty acids <1% of total energy intake.	-
	<5 g of salt per day.	-
	30–45 g of fiber per day, from wholegrain products, fruits and vegetables.	-
	200 g of fruit per day (2–3 servings).	-
	200 g of vegetables per day (2–3 servings)	-
	Fish at least twice a week, one being oily fish.	-
	Consumption of alcoholic beverages should be limited to 2 glasses per day (20 g/day of alcohol) for men and 1 glass per day (10 g/day of alcohol) for non-pregnant women.	-
	In general, when following the rules for a healthy diet, no dietary supplements are needed.	-
**American College of Cardiology/American Heart Association (2013), [19]**	**LDL-C: Advise adults who would benefit from LDL-C lowering to:**	
	1. Consume a dietary pattern that emphasizes intake of vegetables, fruits, and whole grains; includes low-fat dairy products, poultry, fish, legumes, non-tropical vegetable oils, and nuts; and limits intake of sweets, sugar-sweetened beverages, and red meats. a. Adapt this dietary pattern to appropriate calorie requirements, personal and cultural food preferences, and nutrition therapy for other medical conditions (including diabetes). b. Achieve this pattern by following plans such as the DASH dietary pattern, the USDA Food Pattern, or the AHA Diet.	I A
	2. Aim for a dietary pattern that achieves 5%–6% of calories from saturated fat.	I A
	3. Reduce percent of calories from saturated fat	I A
	4. Reduce percent of calories from *trans* fat.	I A
	**BP: Advise adults who would benefit from BP lowering to:**	
	1. Consume a dietary pattern that emphasizes intake of vegetables, fruits, and whole grains; includes low-fat dairy products, poultry, fish, legumes, non-tropical vegetable oils, and nuts; and limits intake of sweets, sugar-sweetened beverages, and red meats. a. Adapt this dietary pattern to appropriate calorie requirements, personal and cultural food preferences, and nutrition therapy for other medical conditions (including diabetes). b. Achieve this pattern by following plans such as the DASH dietary pattern, the USDA Food Pattern, or the AHA Diet.	I A
	2. Lower sodium intake.	I A
	3. Specifically: a. Consume no more than 2400 mg of sodium/day; b. Further reduction of sodium intake to 1500 mg/day can result in even greater reduction in BP; and c. Even without achieving these goals, reducing sodium intake by at least 1000 mg/day lowers BP.	IIa B
	4. Combine the DASH dietary pattern with lower sodium intake.	I A

ACC, American College of Cardiology; AHA, American Heart Association; BMI, body mass index; COR, class of recommendation (I: recommended/indicated; IIa: should be considered); CVD, cardiovascular disease; DASH, dietary approach to Stop Hypertension; ESC, European Society of Cardiology; LDL-C, low density lipoprotein cholesterol; LOE, level of evidence (A: data derived from multiple randomized clinical trials or meta-analyses; B: data derived from a single randomized clinical trial or large non-randomized studies); PUFA, Polyunsaturated Fatty Acids; USDA, United States Department of Agriculture.

**Table 4:** ESC and ACC/AHA dietary recommendations for secondary prevention of CAD.

Society	Diet Recommendations for CAD—Secondary Prevention	LOE
**European Society of Cardiology (2013), [36]**	Energy intake should be limited to the amount of energy needed to maintain (or obtain) a healthy weight (BMI < 25 kg/m^2^).	-
	Saturated fatty acids to account for <10% of total energy intake, through replacement by PUFA.	-
	*Trans* unsaturated fatty acids <1% of total energy intake.	-
	<5 g of salt per day.	-
	30–45 g of fiber per day, from wholegrain products, fruits and vegetables.	-
	200 g of fruit per day (2–3 servings).	-
	200 g of vegetables per day (2–3 servings)	-
	Fish at least twice a week, one being oily fish.	-
	Consumption of alcoholic beverages should be limited to 2 glasses per day (20 g/day of alcohol) for men and 1 glass per day (10 g/day of alcohol) for non-pregnant women.	-
**American College of Cardiology/American Heart Association (2012), [37]**	Dietary therapy for all patients should include reduced intake of saturated fats (to <7% of total calories), *trans* fatty acids (to <1% of total calories), and cholesterol (to <200 mg/day)	B
	All patients should be counseled about the need for lifestyle modification: weight control; increased physical activity; alcohol moderation; sodium reduction; and emphasis on increased consumption of fresh fruits, vegetables, and low-fat dairy products	B
	BMI and/or waist circumference should be assessed at every visit, and the clinician should consistently encourage weight maintenance or reduction through an appropriate balance of lifestyle physical activity, structured exercise, caloric intake, and formal behavioral programs when indicated to maintain or achieve a BMI between 18.5 and 24.9 kg/m^2^ and a waist circumference less than 102 cm (40 inches) in men and less than 88 cm (35 inches) in women (less for certain racial groups)	B
	In patients with symptomatic ischemic heart disease who use alcohol, it might be reasonable for non-pregnant women to have 1 drink (4 ounces of wine, 12 ounces of beer, or 1 ounce of spirits) a day and for men to have 1 or 2 drinks a day, unless alcohol is contraindicated (such as in patients with a history of alcohol abuse or dependence or with liver disease).	C

ACC, American College of Cardiology; AHA, American Heart Association; BMI, body mass index; CAD, coronary artery disease; ESC, European Society of Cardiology; LOE, level of evidence (B: data derived from a single randomized clinical trial or large non-randomized studies; C: consensus of opinion of the experts and/or small studies, retrospective studies, registries); PUFA, Polyunsaturated Fatty Acids.

**Table 5:** ESC and ACC/AHA dietary recommendations for HF.

Society	Diet Recommendations for HF	COR/LOE
**European Society of Cardiology 2012 [45]**	An ω-3 PUFA preparation may be considered to reduce the risk of death and the risk of cardiovascular hospitalization in patients treated with an angiotensin converting enzyme inhibitor (or angiotensin receptor blocker), beta-blocker, and an mineral corticoid receptor antagonist (or angiotensin receptor blocker).	IIb B
	Avoid excessive fluid intake: fluid restriction of 1.5–2 L/day may be considered in patients with severe HF to relieve symptoms and congestion. Restriction of hypotonic fluids may improve hyponatremia. Routine fluid restriction in all patients with mild to moderate symptoms is probably not of benefit. Weight-based fluid restriction (30 mL/kg body weight, 35 mL/kg if body weight >85 kg) may cause less thirst	-
	Monitor and prevent malnutrition.	-
	Eat healthily and keep a healthy weight.	-
	Modest intake of alcohol: abstinence is recommended in patients with alcohol-induced cardiomyopathy. Otherwise, normal alcohol guidelines apply (2 units per day in men or 1 unit per day in women). Note: 1 unit is 10 mL of pure alcohol (e.g., 1 glass of wine, 1/2 pint of beer, 1 measure of spirit).	-
	Sodium restriction may help control the symptoms and signs of congestion in patients with symptomatic HF classes III and IV.	-
**American College of Cardiology/American Heart Association (2013) [4]**	STAGE A: hypertension and lipid disorders should be controlled in accordance with contemporary guidelines to lower the risk of HF.	I A
	STAGE B: in patients with structural cardiac abnormalities, including left ventricular hypertrophy, in the absence of a history of MI or acute coronary syndrome, BP should be controlled in accordance with clinical practice guidelines for hypertension to prevent symptomatic HF	I A
	STAGE C: sodium restriction is reasonable for patients with symptomatic HF to reduce congestive symptoms.	IIa C
	STAGE C: ω-3 PUFA supplementation is reasonable to use as adjunctive therapy in patients with NYHA class II–IV symptoms and HFrEF or HFpEF, unless contraindicated, to reduce mortality and cardiovascular hospitalizations.	IIa B
	STAGE C: nutritional supplements as treatment for HF are not recommended in patients with current or prior symptoms of HFrEF.	III B
	STAGE C: Routine use of nutritional supplements is not recommended for patients with HFpEF.	III C
	STAGE D: fluid restriction (1.5 to 2 L/day) is reasonable in stage D, especially in patients with hyponatremia, to reduce congestive symptoms.	IIa C

ACC, American College of Cardiology; AHA, American Heart Association; BP, blood pressure; COR, class of recommendation (I: recommended/indicated; IIa: should be considered; IIb may be considered; III: not recommended); ESC, European Society of Cardiology; HF, heart failure; HFpEF, heart failure with preserved ejection fraction; HFrEF, heart failure with reduced ejection fraction; LOE, level of evidence (A: data derived from multiple randomized clinical trials or meta-analyses; B: data derived from a single randomized clinical trial or large non-randomized studies; C: consensus of opinion of the experts and/or small studies, retrospective studies, registries); MI, myocardial infarction; NYHA, New York Heart Association; PUFA, polyunsaturated fatty acids.

## Abbreviations

The following abbreviations have been used in the text:

AARP	American Association of Retired Persons
ACC	American College of Cardiology
AER	Albumin Excretion Rate
AHA	American Heart Association
β2-AR	Beta 2 Adrenergic Receptor
BMI	Body Mass Index
BP	Blood Pressure
CAD	Coronary Artery Disease
CARDIA	Coronary Artery Risk Development in Young Adults
CI	Confidence Interval
COR	Class Of Recommendation
CV	Cardiovascular
CVD	Cardiovascular Disease
DALY	Disability-Adjusted Life Year
DASH	Dietary Approach to Stop Hypertension
DBP	Diastolic Blood Pressure
EPIC	European Prospective Investigation into Cancer and nutrition
ESC	European Society of Cardiology
FFQ	Food Frequency Questionnaire
GLYNDIET	Glycemic Index of the Diet study
HALE	Healthy Ageing: a Longitudinal study in Europe
HDL (-C)	High Density Lipoprotein (Cholesterol)
HF	Heart Failure
HFpEF	Heart Failure with preserved Ejection Fraction
HFrEF	Heart Failure with reduced Ejection Fraction
HR	Hazard Ratio
LDL (-C)	Low Density Lipoprotein (Cholesterol)
LOE	Level of Evidence
LVEF	Left Ventricular Ejection Fraction
MED	Mediterranean
MESA	Multi-Ethnic Study of Atherosclerosis
MI	Myocardial Infarction
MLXIPL	Max-Like protein X Interacting Protein-Like
ND	Nordic Diet
NIH	National Institutes of Health
NT-proBNP	N-terminal fragment of the pro-peptide for Brain Natriuretic Peptide
NYHA	New York Heart Association
OMNI-Heart	Optimal Macro-Nutrient Intake Heart trial
PRA	Plasma Renin Activity
PREDIMED	PREvenciόn con DIetaMEDiterránea
PUFA	Polyunsaturated Fatty Acids
QoL	Quality of Life
RAAS	Renin-Angiotensin-Aldosterone System
RR	Relative Risk
SBP	Systolic Blood Pressure
SCD	Sudden Cardiac Death
TCF7L2	Transcription Factor 7-Like 2
TG	Triglycerides
USDA	United States Department of Agriculture

## References

[1] Mozaffarian D., Benjamin E.J., Go A.S., Arnett D.K., Blaha M.J., Cushman M., Das S.R., de Ferranti S., Despres J.P., Fullerton H.J. (2016). Heart disease and stroke statistics-2016 update: A report from the American Heart Association. Circulation.

[2] Townsend N., Nichols M., Scarborough P., Rayner M. (2015). Cardiovascular disease in Europe 2015: Epidemiological update. Eur. Heart J..

[3] (2015). GBD 2013 Mortality and Causes of Death Collaborators. Global, regional, and national age-sex specific all-cause and cause-specific mortality for 240 causes of death, 1990–2013: A systematic analysis for the global burden of disease study 2013. Lancet.

[4] Yusuf S., Reddy S., Ounpuu S., Anand S. (2001). Global burden of cardiovascular diseases: Part II: Variations in cardiovascular disease by specific ethnic groups and geographic regions and prevention strategies. Circulation.

[5] Bhupathiraju S.N., Tucker K.L. (2011). Coronary heart disease prevention: Nutrients, foods, and dietary patterns. Clin. Chim. Acta.

[6] Witte K.K., Byrom R. (2014). Micronutrients for chronic heart failure: End of the road or path to enlightenment?. JACC Heart Fail..

[7] Konstantinov I.E., Jankovic G.M. (2013). Alexander I. Ignatowski: A pioneer in the study of atherosclerosis. Tex. Heart Inst. J..

[8] Keys A. (1957). Diet and the epidemiology of coronary heart disease. J. Am. Med. Assoc..

[9] Keys A. (1997). Coronary heart disease in seven countries, 1970. Nutrition.

[10] Kromhout D., Menotti A., Bloemberg B., Aravanis C., Blackburn H., Buzina R., Dontas A.S., Fidanza F., Giampaoli S., Jansen A. (1995). Dietary saturated and *trans* fatty acids and cholesterol and 25-year mortality from coronary heart disease: The seven countries study. Prev. Med..

[11] Dyerberg J., Bang H.O. (1979). Lipid metabolism, atherogenesis, and haemostasis in Eskimos: The role of the prostaglandin-3 family. Haemostasis.

[12] Spigoni V., Lombardi C., Cito M., Picconi A., Ridolfi V., Andreoli R., Anelli N., Gnudi L., Goldoni M., Zavaroni I. (2014). *n*-3 PUFA increase bioavailability and function of endothelial progenitor cells. Food Funct..

[13] Page I.H., Stare F.J., Corcoran A.C., Pollack H., Wilkinson C.F. (1957). Atherosclerosis and the fat content of the diet. J. Am. Med. Assoc..

[14] Heidemann C., Schulze M.B., Franco O.H., van Dam R.M., Mantzoros C.S., Hu F.B. (2008). Dietary patterns and risk of mortality from cardiovascular disease, cancer, and all causes in a prospective cohort of women. Circulation.

[15] Mozaffarian D., Ludwig D.S. (2010). Dietary guidelines in the 21st century—A time for food. JAMA.

[16] Trichopoulou A., Costacou T., Bamia C., Trichopoulos D. (2003). Adherence to a Mediterranean diet and survival in a Greek population. N. Engl. J. Med..

[17] Aravanis C., Corcondilas A., Dontas A.S., Lekos D., Keys A. (1970). Coronary heart disease in seven countries. IX. The Greek islands of Crete and Corfu. Circulation.

[18] Fidanza F., Puddu V., Imbimbo A.B., Menotti A., Keys A. (1970). Coronary heart disease in seven countries. VII. Five-year experience in rural Italy. Circulation.

[19] Eckel R.H., Jakicic J.M., Ard J.D., de Jesus J.M., Miller N.H., Hubbard V.S., Lee I.M., Lichtenstein A.H., Loria C.M., Millen B.E. (2014). 2013 AHA/ACC guideline on lifestyle management to reduce cardiovascular risk: A report of the American College of Cardiology/American Heart Association task force on practice guidelines. J. Am. Coll. Cardiol..

[20] Trichopoulou A., Kouris-Blazos A., Wahlqvist M.L., Gnardellis C., Lagiou P., Polychronopoulos E., Vassilakou T., Lipworth L., Trichopoulos D. (1995). Diet and overall survival in elderly people. BMJ.

[21] Kouris-Blazos A., Gnardellis C., Wahlqvist M.L., Trichopoulos D., Lukito W., Trichopoulou A. (1999). Are the advantages of the Mediterranean diet transferable to other populations? A cohort study in Melbourne, Australia. Br. J. Nutr..

[22] Osler M., Schroll M. (1997). Diet and mortality in a cohort of elderly people in a north European community. Int. J. Epidemiol..

[23] Lasheras C., Fernandez S., Patterson A.M. (2000). Mediterranean diet and age with respect to overall survival in institutionalized, nonsmoking elderly people. Am. J. Clin. Nutr..

[24] Trichopoulou A., Bamia C., Trichopoulos D. (2009). Anatomy of health effects of Mediterranean diet: Greek EPIC prospective cohort study. BMJ.

[25] Knoops K.T., de Groot L.C., Kromhout D., Perrin A.E., Moreiras-Varela O., Menotti A., van Staveren W.A. (2004). Mediterranean diet, lifestyle factors, and 10-year mortality in elderly European men and women: The hale project. JAMA.

[26] Mitrou P.N., Kipnis V., Thiebaut A.C., Reedy J., Subar A.F., Wirfalt E., Flood A., Mouw T., Hollenbeck A.R., Leitzmann M.F. (2007). Mediterranean dietary pattern and prediction of all-cause mortality in a US population: Results from the NIH–AARP diet and health study. Arch. Intern. Med..

[27] Fung T.T., Rexrode K.M., Mantzoros C.S., Manson J.E., Willett W.C., Hu F.B. (2009). Mediterranean diet and incidence of and mortality from coronary heart disease and stroke in women. Circulation.

[28] Perk J., de Backer G., Gohlke H., Graham I., Reiner Z., Verschuren M., Albus C., Benlian P., Boysen G., Cifkova R. (2012). European guidelines on cardiovascular disease prevention in clinical practice (version 2012). The fifth joint task force of the European society of cardiology and other societies on cardiovascular disease prevention in clinical practice (constituted by representatives of nine societies and by invited experts). Eur. Heart. J..

[29] Estruch R., Ros E., Salas-Salvado J., Covas M.I., Corella D., Aros F., Gomez-Gracia E., Ruiz-Gutierrez V., Fiol M., Lapetra J. (2013). Primary prevention of cardiovascular disease with a Mediterranean diet. N. Engl. J. Med..

[30] Appel L.J., van Horn L. (2013). Did the predimed trial test a Mediterranean diet?. N. Engl. J. Med..

[31] De Lorgeril M., Renaud S., Mamelle N., Salen P., Martin J.L., Monjaud I., Guidollet J., Touboul P., Delaye J. (1994). Mediterranean alpha-linolenic acid-rich diet in secondary prevention of coronary heart disease. Lancet.

[32] De Lorgeril M., Salen P., Martin J.L., Monjaud I., Delaye J., Mamelle N. (1999). Mediterranean diet, traditional risk factors, and the rate of cardiovascular complications after myocardial infarction: Final report of the lyon diet heart study. Circulation.

[33] Booth J.N., Levitan E.B., Brown T.M., Farkouh M.E., Safford M.M., Muntner P. (2014). Effect of sustaining lifestyle modifications (nonsmoking, weight reduction, physical activity, and mediterranean diet) after healing of myocardial infarction, percutaneous intervention, or coronary bypass (from the reasons for geographic and racial differences in stroke study). Am. J. Cardiol..

[34] Lopez-Garcia E., Rodriguez-Artalejo F., Li T.Y., Fung T.T., Li S., Willett W.C., Rimm E.B., Hu F.B. (2014). The mediterranean-style dietary pattern and mortality among men and women with cardiovascular disease. Am. J. Clin. Nutr..

[35] Tuttle K.R., Shuler L.A., Packard D.P., Milton J.E., Daratha K.B., Bibus D.M., Short R.A. (2008). Comparison of low-fat versus mediterranean-style dietary intervention after first myocardial infarction (from the heart institute of spokane diet intervention and evaluation trial). Am. J. Cardiol..

[36] Force M.T., Montalescot G., Sechtem U., Achenbach S., Andreotti F., Arden C., Budaj A., Bugiardini R., Crea F., Cuisset T. (2013). 2013 ESC guidelines on the management of stable coronary artery disease: The task force on the management of stable coronary artery disease of the European Society of Cardiology. Eur. Heart J..

[37] Fihn S.D., Gardin J.M., Abrams J., Berra K., Blankenship J.C., Dallas A.P., Douglas P.S., Foody J.M., Gerber T.C., Hinderliter A.L. (2012). 2012 ACCF/AHA/ACP/AATS/PCNA/SCAI/STS guideline for the diagnosis and management of patients with stable ischemic heart disease: A report of the American College of Cardiology Foundation/American Heart Association task force on practice guidelines, and the American College of Physicians, American Association for Thoracic Surgery, Preventive Cardiovascular Nurses Association, Society for Cardiovascular Angiography and Interventions, and Society of Thoracic Surgeons. J. Am. Coll. Cardiol..

[38] Metra M., Zaca V., Parati G., Agostoni P., Bonadies M., Ciccone M., Cas A.D., Iacoviello M., Lagioia R., Lombardi C. (2011). Cardiovascular and noncardiovascular comorbidities in patients with chronic heart failure. J. Cardiovasc. Med. (Hagerstown).

[39] Lazzarini V., Bettari L., Bugatti S., Carubelli V., Lombardi C., Metra M., dei Cas L. (2012). Can we prevent or treat renal dysfunction in acute heart failure?. Heart Fail. Rev..

[40] Carubelli V., Metra M., Lombardi C., Bettari L., Bugatti S., Lazzarini V., dei Cas L. (2012). Renal dysfunction in acute heart failure: Epidemiology, mechanisms and assessment. Heart Fail. Rev..

[41] Braunwald E. (2015). The war against heart failure: The lancet lecture. Lancet.

[42] Metra M., Bugatti S., Bettari L., Carubelli V., Danesi R., Lazzarini V., Lombardi C., Cas L.D. (2011). Can we improve the treatment of congestion in heart failure?. Expert Opin. Pharmacother..

[43] Metra M., Bettari L., Carubelli V., Bugatti S., dei Cas A., del Magro F., Lazzarini V., Lombardi C., dei Cas L. (2011). Use of inotropic agents in patients with advanced heart failure: Lessons from recent trials and hopes for new agents. Drugs.

[44] Castrini A.I., Carubelli V., Lazzarini V., Bonadei I., Lombardi C., Metra M. (2015). Serelaxin a novel treatment for acute heart failure. Expert Rev. Clin. Pharmacol..

[45] McMurray J.J., Adamopoulos S., Anker S.D., Auricchio A., Böhm M., Dickstein K., Falk V., Filippatos G., Fonseca C., Gomez-Sanchez M.A. (2012). Esc guidelines for the diagnosis and treatment of acute and chronic heart failure 2012: The task force for the diagnosis and treatment of acute and chronic heart failure 2012 of the European Society of Cardiology. Developed in collaboration with the Heart Failure Association (HFA) of the ESC. Eur. J. Heart Fail..

[46] Yancy C.W., Jessup M., Bozkurt B., Butler J., Casey D.E., Drazner M.H., Fonarow G.C., Geraci S.A., Horwich T., Januzzi J.L. (2013). 2013 ACCF/AHA guideline for the management of heart failure: A report of the American College of Cardiology Foundation/American Heart Association task force on practice guidelines. J. Am. Coll. Cardiol..

[47] Braunwald E. (2013). Heart failure. JACC Heart Fail..

[48] Nodari S., Manerba A., Vaccari A., Milesi G., Carubelli V., Lazzarini V., Lombardi C., Ettori F., Metra M., dei Cas A. (2012). Six-year prognosis of diabetic patients with coronary artery disease. Eur. J. Clin. Invest..

[49] Nordmann A.J., Suter-Zimmermann K., Bucher H.C., Shai I., Tuttle K.R., Estruch R., Briel M. (2011). Meta-analysis comparing mediterranean to low–fat diets for modification of cardiovascular risk factors. Am. J. Med..

[50] Fung T.T., McCullough M.L., Newby P.K., Manson J.E., Meigs J.B., Rifai N., Willett W.C., Hu F.B. (2005). Diet-quality scores and plasma concentrations of markers of inflammation and endothelial dysfunction. Am. J. Clin. Nutr..

[51] Fito M., Guxens M., Corella D., Saez G., Estruch R., de la Torre R., Frances F., Cabezas C., Mdel C.L.-S., Marrugat J. (2007). Effect of a traditional mediterranean diet on lipoprotein oxidation: A randomized controlled trial. Arch. Intern. Med..

[52] Tektonidis T.G., Akesson A., Gigante B., Wolk A., Larsson S.C. (2016). Adherence to a mediterranean diet is associated with reduced risk of heart failure in men. Eur. J. Heart Fail..

[53] Tektonidis T.G., Akesson A., Gigante B., Wolk A., Larsson S.C. (2015). A mediterranean diet and risk of myocardial infarction, heart failure and stroke: A population-based cohort study. Atherosclerosis.

[54] Chrysohoou C., Panagiotakos D.B., Aggelopoulos P., Kastorini C.M., Kehagia I., Pitsavos C., Stefanadis C. (2010). The mediterranean diet contributes to the preservation of left ventricular systolic function and to the long-term favorable prognosis of patients who have had an acute coronary event. Am. J. Clin. Nutr..

[55] Chrysohoou C., Pitsavos C., Metallinos G., Antoniou C., Oikonomou E., Kotroyiannis I., Tsantilas A., Tsitsinakis G., Tousoulis D., Panagiotakos D.B. (2012). Cross-sectional relationship of a mediterranean type diet to diastolic heart function in chronic heart failure patients. Heart Vessels.

[56] Fito M., Estruch R., Salas-Salvado J., Martinez-Gonzalez M.A., Aros F., Vila J., Corella D., Diaz O., Saez G., de la Torre R. (2014). Effect of the mediterranean diet on heart failure biomarkers: A randomized sample from the predimed trial. Eur. J. Heart Fail..

[57] Bertoia M.L., Triche E.W., Michaud D.S., Baylin A., Hogan J.W., Neuhouser M.L., Tinker L.F., van Horn L., Waring M.E., Li W. (2014). Mediterranean and dietary approaches to stop hypertension dietary patterns and risk of sudden cardiac death in postmenopausal women. Am. J. Clin. Nutr..

[58] Chiuve S.E., Fung T.T., Rexrode K.M., Spiegelman D., Manson J.E., Stampfer M.J., Albert C.M. (2011). Adherence to a low-risk, healthy lifestyle and risk of sudden cardiac death among women. JAMA.

[59] Banke A., Schou M., Videbaek L., Moller J.E., Torp-Pedersen C., Gustafsson F., Dahl J.S., Kober L., Hildebrandt P.R., Gislason G.H. (2016). Incidence of cancer in patients with chronic heart failure: A long–term follow-up study. Eur. J. Heart Fail..

[60] Sofi F., Macchi C., Abbate R., Gensini G.F., Casini A. (2014). Mediterranean diet and health status: An updated meta-analysis and a proposal for a literature-based adherence score. Public Health Nutr..

[61] Diaz-Lopez A., Babio N., Martinez-Gonzalez M.A., Corella D., Amor A.J., Fito M., Estruch R., Aros F., Gomez-Gracia E., Fiol M. (2015). Mediterranean diet, retinopathy, nephropathy, and microvascular diabetes complications: A *post hoc* analysis of a randomized trial. Diabetes Care.

[62] Sorli-Aguilar M., Martin-Lujan F., Santigosa-Ayala A., Pinol-Moreso J.L., Flores-Mateo G., Basora-Gallisa J., Arija-Val V., Sola-Alberich R. (2015). Effects of mediterranean diet on lung function in smokers: A randomised, parallel and controlled protocol. BMC Public Health.

[63] Levitan E.B., Lewis C.E., Tinker L.F., Eaton C.B., Ahmed A., Manson J.E., Snetselaar L.G., Martin L.W., Trevisan M., Howard B.V. (2013). Mediterranean and dash diet scores and mortality in women with heart failure: The women’s health initiative. Circ. Heart Fail..

[64] Soukoulis V., Dihu J.B., Sole M., Anker S.D., Cleland J., Fonarow G.C., Metra M., Pasini E., Strzelczyk T., Taegtmeyer H. (2009). Micronutrient deficiencies an unmet need in heart failure. J. Am. Coll. Cardiol..

[65] Carubelli V., Castrini A.I., Lazzarini V., Gheorghiade M., Metra M., Lombardi C. (2015). Amino acids and derivatives, a new treatment of chronic heart failure?. Heart Fail. Rev..

[66] Lombardi C., Carubelli V., Lazzarini V., Vizzardi E., Bordonali T., Ciccarese C., Castrini A.I., dei Cas A., Nodari S., Metra M. (2015). Effects of oral administration of orodispersible levo-carnosine on quality of life and exercise performance in patients with chronic heart failure. Nutrition.

[67] Lombardi C., Carubelli V., Lazzarini V., Vizzardi E., Quinzani F., Guidetti F., Rovetta R., Nodari S., Gheorghiade M., Metra M. (2014). Effects of oral amino acid supplements on functional capacity in patients with chronic heart failure. Clin. Med. Insights. Cardiol..

[68] Bach-Faig A., Berry E.M., Lairon D., Reguant J., Trichopoulou A., Dernini S., Medina F.X., Battino M., Belahsen R., Miranda G. (2011). Mediterranean diet pyramid today. Science and cultural updates. Public Health Nutr..

[69] Burt V.L., Whelton P., Roccella E.J., Brown C., Cutler J.A., Higgins M., Horan M.J., Labarthe D. (1995). Prevalence of hypertension in the us adult population. Results from the third national health and nutrition examination survey, 1988–1991. Hypertension.

[70] Sacks F.M., Rosner B., Kass E.H. (1974). Blood pressure in vegetarians. Am. J. Epidemiol..

[71] Appel L.J., Moore T.J., Obarzanek E., Vollmer W.M., Svetkey L.P., Sacks F.M., Bray G.A., Vogt T.M., Cutler J.A., Windhauser M.M. (1997). A clinical trial of the effects of dietary patterns on blood pressure. Dash collaborative research group. N. Engl. J. Med..

[72] Obarzanek E., Sacks F.M., Vollmer W.M., Bray G.A., Miller E.R., Lin P.H., Karanja N.M., Most-Windhauser M.M., Moore T.J., Swain J.F. (2001). Effects on blood lipids of a blood pressure-lowering diet: The dietary approaches to stop hypertension (DASH) trial. Am. J. Clin. Nutr..

[73] Svetkey L.P., Simons-Morton D., Vollmer W.M., Appel L.J., Conlin P.R., Ryan D.H., Ard J., Kennedy B.M. (1999). Effects of dietary patterns on blood pressure: Subgroup analysis of the dietary approaches to stop hypertension (DASH) randomized clinical trial. Arch. Intern. Med..

[74] Ard J.D., Coffman C.J., Lin P.H., Svetkey L.P. (2004). One-year follow-up study of blood pressure and dietary patterns in dietary approaches to stop hypertension (DASH)-sodium participants. Am. J. Hypertens..

[75] Jacobs D.R., Gross M.D., Steffen L., Steffes M.W., Yu X., Svetkey L.P., Appel L.J., Vollmer W.M., Bray G.A., Moore T. (2009). The effects of dietary patterns on urinary albumin excretion: Results of the dietary approaches to stop hypertension (DASH) trial. Am. J. Kidney Dis..

[76] Chang A., van Horn L., Jacobs D.R., Liu K., Muntner P., Newsome B., Shoham D.A., Durazo-Arvizu R., Bibbins-Domingo K., Reis J. (2013). Lifestyle-related factors, obesity, and incident microalbuminuria: The cardia (coronary artery risk development in young adults) study. Am. J. Kidney. Dis..

[77] Svetkey L.P., Sacks F.M., Obarzanek E., Vollmer W.M., Appel L.J., Lin P.H., Karanja N.M., Harsha D.W., Bray G.A., Aickin M. (1999). The DASH diet, sodium intake and blood pressure trial (DASH-sodium): Rationale and design. DASH-sodium collaborative research group. J. Am. Diet. Assoc..

[78] Sacks F.M., Svetkey L.P., Vollmer W.M., Appel L.J., Bray G.A., Harsha D., Obarzanek E., Conlin P.R., Miller E.R., Simons-Morton D.G. (2001). Effects on blood pressure of reduced dietary sodium and the dietary approaches to stop hypertension (DASH) diet. DASH-sodium collaborative research group. N. Engl. J. Med..

[79] Carey V.J., Bishop L., Charleston J., Conlin P., Erlinger T., Laranjo N., McCarron P., Miller E., Rosner B., Swain J. (2005). Rationale and design of the optimal macro-nutrient intake heart trial to prevent heart disease (omni-heart). Clin. Trials.

[80] Appel L.J., Sacks F.M., Carey V.J., Obarzanek E., Swain J.F., Miller E.R., Conlin P.R., Erlinger T.P., Rosner B.A., Laranjo N.M. (2005). Effects of protein, monounsaturated fat, and carbohydrate intake on blood pressure and serum lipids: Results of the omniheart randomized trial. JAMA.

[81] Conlin P.R., Erlinger T.P., Bohannon A., Miller E.R., Appel L.J., Svetkey L.P., Moore T.J. (2003). The DASH diet enhances the blood pressure response to losartan in hypertensive patients. Am. J. Hypertens..

[82] Kirpizidis H., Stavrati A., Geleris P. (2005). Assessment of quality of life in a randomized clinical trial of candesartan only or in combination with DASH diet for hypertensive patients. J. Cardiol..

[83] Aljefree N., Ahmed F. (2015). Association between dietary pattern and risk of cardiovascular disease among adults in the middle east and north Africa region: A systematic review. Food Nutr. Res..

[84] Fung T.T., Chiuve S.E., McCullough M.L., Rexrode K.M., Logroscino G., Hu F.B. (2008). Adherence to a DASH-style diet and risk of coronary heart disease and stroke in women. Arch. Intern. Med..

[85] Mokhtari Z., Hosseini S., Miri R., Baghestani A.R., Zahedirad M., Rismanchi M., Nasrollahzadeh J. (2015). Relationship between dietary approaches to stop hypertension score and alternative healthy eating index score with plasma asymmetrical dimethylarginine levels in patients referring for coronary angiography. J. Hum. Nutr. Diet..

[86] Appel L.J., Champagne C.M., Harsha D.W., Cooper L.S., Obarzanek E., Elmer P.J., Stevens V.J., Vollmer W.M., Lin P.H., Svetkey L.P. (2003). Effects of comprehensive lifestyle modification on blood pressure control: Main results of the premier clinical trial. JAMA.

[87] Parikh A., Lipsitz S.R., Natarajan S. (2009). Association between a DASH-like diet and mortality in adults with hypertension: Findings from a population-based follow-up study. Am. J. Hypertens..

[88] Levitan E.B., Wolk A., Mittleman M.A. (2009). Consistency with the DASH diet and incidence of heart failure. Arch. Intern. Med..

[89] Levitan E.B., Wolk A., Mittleman M.A. (2009). Relation of consistency with the dietary approaches to stop hypertension diet and incidence of heart failure in men aged 45 to 79 years. Am. J. Cardiol..

[90] Salehi-Abargouei A., Maghsoudi Z., Shirani F., Azadbakht L. (2013). Effects of dietary approaches to stop hypertension (DASH)-style diet on fatal or nonfatal cardiovascular diseases—Incidence: A systematic review and meta-analysis on observational prospective studies. Nutrition.

[91] Komajda M., Lam C.S. (2014). Heart failure with preserved ejection fraction: A clinical dilemma. Eur. Heart J..

[92] Hummel S.L., Seymour E.M., Brook R.D., Kolias T.J., Sheth S.S., Rosenblum H.R., Wells J.M., Weder A.B. (2012). Low-sodium dietary approaches to stop hypertension diet reduces blood pressure, arterial stiffness, and oxidative stress in hypertensive heart failure with preserved ejection fraction. Hypertension.

[93] Hummel S.L., Seymour E.M., Brook R.D., Sheth S.S., Ghosh E., Zhu S., Weder A.B., Kovacs S.J., Kolias T.J. (2013). Low-sodium DASH diet improves diastolic function and ventricular-arterial coupling in hypertensive heart failure with preserved ejection fraction. Circ. Heart Fail..

[94] Mathew A.V., Seymour E.M., Byun J., Pennathur S., Hummel S.L. (2015). Altered metabolic profile with sodium-restricted dietary approaches to stop hypertension diet in hypertensive heart failure with preserved ejection fraction. J. Card. Fail..

[95] Nguyen H.T., Bertoni A.G., Nettleton J.A., Bluemke D.A., Levitan E.B., Burke G.L. (2012). DASH eating pattern is associated with favorable left ventricular function in the multi-ethnic study of atherosclerosis. J. Am. Coll. Nutr..

[96] Rifai L., Pisano C., Hayden J., Sulo S., Silver M.A. (2015). Impact of the DASH diet on endothelial function, exercise capacity, and quality of life in patients with heart failure. Proceedings (Bayl. Univ. Med. Cent.).

[97] Akita S., Sacks F.M., Svetkey L.P., Conlin P.R., Kimura G., Group D.A.-S.T.C.R. (2003). Effects of the dietary approaches to stop hypertension (DASH) diet on the pressure-natriuresis relationship. Hypertension.

[98] Willcox D.C., Willcox B.J., Todoriki H., Suzuki M. (2009). The okinawan diet: Health implications of a low-calorie, nutrient-dense, antioxidant-rich dietary pattern low in glycemic load. J. Am. Coll. Nutr..

[99] Rivas M., Garay R.P., Escanero J.F., Cia P., Cia P., Alda J.O. (2002). Soy milk lowers blood pressure in men and women with mild to moderate essential hypertension. J. Nutr..

[100] Mori T.A. (2014). Dietary *n*-3 PUFA and CVD: A review of the evidence. Proc. Nutr. Soc..

[101] Kuriyama S., Shimazu T., Ohmori K., Kikuchi N., Nakaya N., Nishino Y., Tsubono Y., Tsuji I. (2006). Green tea consumption and mortality due to cardiovascular disease, cancer, and all causes in Japan: The ohsaki study. JAMA.

[102] Shimazu T., Kuriyama S., Hozawa A., Ohmori K., Sato Y., Nakaya N., Nishino Y., Tsubono Y., Tsuji I. (2007). Dietary patterns and cardiovascular disease mortality in Japan: A prospective cohort study. Int. J. Epidemiol..

[103] Niu K., Momma H., Kobayashi Y., Guan L., Chujo M., Otomo A., Ouchi E., Nagatomi R. (2016). The traditional Japanese dietary pattern and longitudinal changes in cardiovascular disease risk factors in apparently healthy Japanese adults. Eur. J. Nutr..

[104] Adamsson V., Reumark A., Cederholm T., Vessby B., Riserus U., Johansson G. (2012). What is a healthy nordic diet? Foods and nutrients in the nordiet study. Food Nutr. Res..

[105] Adamsson V., Reumark A., Fredriksson I.B., Hammarstrom E., Vessby B., Johansson G., Riserus U. (2011). Effects of a healthy nordic diet on cardiovascular risk factors in hypercholesterolaemic subjects: A randomized controlled trial (nordiet). J. Intern. Med..

[106] Poulsen S.K., Due A., Jordy A.B., Kiens B., Stark K.D., Stender S., Holst C., Astrup A., Larsen T.M. (2014). Health effect of the new Nordic diet in adults with increased waist circumference: A 6-mo randomized controlled trial. Am. J. Clin. Nutr..

[107] Uusitupa M., Hermansen K., Savolainen M.J., Schwab U., Kolehmainen M., Brader L., Mortensen L.S., Cloetens L., Johansson-Persson A., Onning G. (2013). Effects of an isocaloric healthy nordic diet on insulin sensitivity, lipid profile and inflammation markers in metabolic syndrome—A randomized study (sysdiet). J. Intern. Med..

[108] Olsen A., Egeberg R., Halkjaer J., Christensen J., Overvad K., Tjonneland A. (2011). Healthy aspects of the nordic diet are related to lower total mortality. J. Nutr..

[109] Roswall N., Sandin S., Lof M., Skeie G., Olsen A., Adami H.O., Weiderpass E. (2015). Adherence to the healthy nordic food index and total and cause-specific mortality among Swedish women. Eur. J. Epidemiol..

[110] Roswall N., Li Y., Kyro C., Sandin S., Lof M., Adami H.O., Weiderpass E. (2015). No association between adherence to a healthy nordic food index and colorectal cancer: Results from a Swedish cohort study. Cancer Epidemiol. Biomark. Prev..

[111] Riserus U. (2015). Healthy nordic diet and cardiovascular disease. J. Intern. Med..

[112] Wang F., Zheng J., Yang B., Jiang J., Fu Y., Li D. (2015). Effects of vegetarian diets on blood lipids: A systematic review and meta-analysis of randomized controlled trials. J. Am. Heart Assoc..

[113] Dinu M., Abbate R., Gensini G.F., Casini A., Sofi F. (2016). Vegetarian, vegan diets and multiple health outcomes: A systematic review with meta-analysis of observational studies. Crit. Rev. Food Sci. Nutr..

[114] Key T.J., Fraser G.E., Thorogood M., Appleby P.N., Beral V., Reeves G., Burr M.L., Chang-Claude J., Frentzel-Beyme R., Kuzma J.W. (1999). Mortality in vegetarians and nonvegetarians: Detailed findings from a collaborative analysis of 5 prospective studies. Am. J. Clin. Nutr..

[115] Appleby P.N., Crowe F.L., Bradbury K.E., Travis R.C., Key T.J. (2016). Mortality in vegetarians and comparable nonvegetarians in the United Kingdom. Am. J. Clin. Nutr..

[116] Jenkins D.J., Kendall C.W., Mehling C.C., Parker T., Rao A.V., Agarwal S., Novokmet R., Jones P.J., Raeini M., Story J.A. (1999). Combined effect of vegetable protein (soy) and soluble fiber added to a standard cholesterol-lowering diet. Metabolism.

[117] Jenkins D.J., Josse A.R., Wong J.M., Nguyen T.H., Kendall C.W. (2007). The portfolio diet for cardiovascular risk reduction. Curr. Atheroscler. Rep..

[118] Jenkins D.J., Kendall C.W., Marchie A., Faulkner D.A., Wong J.M., de Souza R., Emam A., Parker T.L., Vidgen E., Lapsley K.G. (2003). Effects of a dietary portfolio of cholesterol-lowering foods *vs* lovastatin on serum lipids and c-reactive protein. JAMA.

[119] Jenkins D.J., Jones P.J., Frohlich J., Lamarche B., Ireland C., Nishi S.K., Srichaikul K., Galange P., Pellini C., Faulkner D. (2015). The effect of a dietary portfolio compared to a DASH-type diet on blood pressure. Nutr. Metab. Cardiovasc. Dis..

[120] Keith M., Kuliszewski M.A., Liao C., Peeva V., Ahmed M., Tran S., Sorokin K., Jenkins D.J., Errett L., Leong-Poi H. (2015). A modified portfolio diet complements medical management to reduce cardiovascular risk factors in diabetic patients with coronary artery disease. Clin. Nutr..

[121] Jenkins D.J., Wolever T.M., Taylor R.H., Barker H., Fielden H., Baldwin J.M., Bowling A.C., Newman H.C., Jenkins A.L., Goff D.V. (1981). Glycemic index of foods: A physiological basis for carbohydrate exchange. Am. J. Clin. Nutr..

[122] Ma X.Y., Liu J.P., Song Z.Y. (2012). Glycemic load, glycemic index and risk of cardiovascular diseases: Meta-analyses of prospective studies. Atherosclerosis.

[123] Dong J.Y., Zhang Y.H., Wang P., Qin L.Q. (2012). Meta-analysis of dietary glycemic load and glycemic index in relation to risk of coronary heart disease. Am. J. Cardiol..

[124] Sacks F.M., Carey V.J., Anderson C.A., Miller E.R., Copeland T., Charleston J., Harshfield B.J., Laranjo N., McCarron P., Swain J. (2014). Effects of high *vs* low glycemic index of dietary carbohydrate on cardiovascular disease risk factors and insulin sensitivity: The omnicarb randomized clinical trial. JAMA.

[125] Levitan E.B., Mittleman M.A., Wolk A. (2010). Dietary glycemic index, dietary glycemic load, and incidence of heart failure events: A prospective study of middle-aged and elderly women. J. Am. Coll. Nutr..

[126] Juanola-Falgarona M., Salas-Salvado J., Ibarrola-Jurado N., Rabassa-Soler A., Diaz-Lopez A., Guasch-Ferre M., Hernandez-Alonso P., Balanza R., Bullo M. (2014). Effect of the glycemic index of the diet on weight loss, modulation of satiety, inflammation, and other metabolic risk factors: A randomized controlled trial. Am. J. Clin. Nutr..

[127] Arnold J.M., Liu P., Demers C., Dorian P., Giannetti N., Haddad H., Heckman G.A., Howlett J.G., Ignaszewski A., Johnstone D.E. (2006). Canadian cardiovascular society consensus conference recommendations on heart failure 2006: Diagnosis and management. Can. J. Cardiol..

[128] Elliott P., Stamler J., Nichols R., Dyer A.R., Stamler R., Kesteloot H., Marmot M. (1996). Intersalt revisited: Further analyses of 24 hour sodium excretion and blood pressure within and across populations. Intersalt cooperative research group. BMJ.

[129] Zhou B.F., Stamler J., Dennis B., Moag-Stahlberg A., Okuda N., Robertson C., Zhao L., Chan Q., Elliott P., Group I.R. (2003). Nutrient intakes of middle-aged men and women in China, Japan, United Kingdom, and United States in the late 1990s: The intermap study. J. Hum. Hypertens..

[130] Cook N.R., Cutler J.A., Obarzanek E., Buring J.E., Rexrode K.M., Kumanyika S.K., Appel L.J., Whelton P.K. (2007). Long term effects of dietary sodium reduction on cardiovascular disease outcomes: Observational follow-up of the trials of hypertension prevention (TOHP). BMJ.

[131] He F.J., MacGregor G.A. (2011). Salt reduction lowers cardiovascular risk: Meta-analysis of outcome trials. Lancet.

[132] Hooper L., Bartlett C., Davey Smith G., Ebrahim S. (2002). Systematic review of long term effects of advice to reduce dietary salt in adults. BMJ.

[133] Taylor R.S., Ashton K.E., Moxham T., Hooper L., Ebrahim S. (2011). Reduced dietary salt for the prevention of cardiovascular disease: A meta-analysis of randomized controlled trials (cochrane review). Am. J. Hypertens..

[134] Song E.K., Moser D.K., Dunbar S.B., Pressler S.J., Lennie T.A. (2014). Dietary sodium restriction below 2 g per day predicted shorter event-free survival in patients with mild heart failure. Eur. J. Cardiovasc. Nurs..

[135] Doukky R., Avery E., Mangla A., Collado F.M., Ibrahim Z., Poulin M.F., Richardson D., Powell L.H. (2016). Impact of dietary sodium restriction on heart failure outcomes. JACC Heart Fail..

[136] Identifier nct02467296, nct02148679, nct01733017, and nct02012179. http://www.ClinicalTrials.gov.

[137] Mellen P.B., Gao S.K., Vitolins M.Z., Goff D.C. (2008). Deteriorating dietary habits among adults with hypertension: DASH dietary accordance, NHANES 1988–1994 and 1999–2004. Arch. Intern. Med..

[138] Scourboutakos M.J., Semnani-Azad Z., L’Abbe M.R. (2013). Restaurant meals: Almost a full day’s worth of calories, fats, and sodium. JAMA Intern. Med..

[139] Young C.M., Batch B.C., Svetkey L.P. (2008). Effect of socioeconomic status on food availability and cost of the dietary approaches to stop hypertension (DASH) dietary pattern. J. Clin. Hypertens (Greenwich).

[140] Monsivais P., Scarborough P., Lloyd T., Mizdrak A., Luben R., Mulligan A.A., Wareham N.J., Woodcock J. (2015). Greater accordance with the dietary approaches to stop hypertension dietary pattern is associated with lower diet-related greenhouse gas production but higher dietary costs in the United Kingdom. Am. J. Clin. Nutr..

[141] Monsivais P., Rehm C.D., Drewnowski A. (2013). The DASH diet and diet costs among ethnic and racial groups in the United States. JAMA Intern. Med..

[142] Schroder H., Serra-Majem L., Subirana I., Izquierdo-Pulido M., Fito M., Elosua R. (2016). Association of increased monetary cost of dietary intake, diet quality and weight management in Spanish adults. Br. J. Nutr..

[143] Algert S.J., Agrawal A., Lewis D.S. (2006). Disparities in access to fresh produce in low-income neighborhoods in Los Angeles. Am. J. Prev. Med..

[144] Bertoni A.G., Foy C.G., Hunter J.C., Quandt S.A., Vitolins M.Z., Whitt-Glover M.C. (2011). A multilevel assessment of barriers to adoption of dietary approaches to stop hypertension (DASH) among African Americans of low socioeconomic status. J. Health Care Poor Underserved.

[145] Hu E.A., Toledo E., Diez-Espino J., Estruch R., Corella D., Salas-Salvado J., Vinyoles E., Gomez-Gracia E., Aros F., Fiol M. (2013). Lifestyles and risk factors associated with adherence to the mediterranean diet: A baseline assessment of the predimed trial. PLoS ONE.

[146] Wong M.C., Wang H.H., Kwan M.W., Fong B.C., Chan W.M., Zhang D.X., Li S.T., Yan B.P., Coats A.J., Griffiths S.M. (2015). Dietary counselling has no effect on cardiovascular risk factors among Chinese grade 1 hypertensive patients: A randomized controlled trial. Eur. Heart J..

[147] Deutch B., Dyerberg J., Pedersen H.S., Aschlund E., Hansen J.C. (2007). Traditional and modern greenlandic food—Dietary composition, nutrients and contaminants. Sci. Total Environ..

[148] Ziv A., Vogel O., Keret D., Pintov S., Bodenstein E., Wolkomir K., Doenyas K., Mirovski Y., Efrati S. (2013). Comprehensive approach to lower blood pressure (calm-bp): A randomized controlled trial of a multifactorial lifestyle intervention. J. Hum. Hypertens..

[149] Blumenthal J.A., Babyak M.A., Hinderliter A., Watkins L.L., Craighead L., Lin P.H., Caccia C., Johnson J., Waugh R., Sherwood A. (2010). Effects of the DASH diet alone and in combination with exercise and weight loss on blood pressure and cardiovascular biomarkers in men and women with high blood pressure: The encore study. Arch. Intern. Med..

[150] Konstantinidou V., Daimiel L., Ordovas J.M. (2014). Personalized nutrition and cardiovascular disease prevention: From framingham to predimed. Adv. Nutr..

[151] Ordovas J.M. (2006). Genetic interactions with diet influence the risk of cardiovascular disease. Am. J. Clin. Nutr..

[152] Shen J., Arnett D.K., Peacock J.M., Parnell L.D., Kraja A., Hixson J.E., Tsai M.Y., Lai C.Q., Kabagambe E.K., Straka R.J. (2007). Interleukin1beta genetic polymorphisms interact with polyunsaturated fatty acids to modulate risk of the metabolic syndrome. J. Nutr..

[153] Garaulet M., Lee Y.C., Shen J., Parnell L.D., Arnett D.K., Tsai M.Y., Lai C.Q., Ordovas J.M. (2009). Clock genetic variation and metabolic syndrome risk: Modulation by monounsaturated fatty acids. Am. J. Clin. Nutr..

[154] Corella D., Carrasco P., Sorli J.V., Estruch R., Rico-Sanz J., Martinez-Gonzalez M.A., Salas-Salvado J., Covas M.I., Coltell O., Aros F. (2013). Mediterranean diet reduces the adverse effect of the tcf7l2-rs7903146 polymorphism on cardiovascular risk factors and stroke incidence: A randomized controlled trial in a high-cardiovascular-risk population. Diabetes Care.

[155] Palmer N.D., Hester J.M., An S.S., Adeyemo A., Rotimi C., Langefeld C.D., Freedman B.I., Ng M.C., Bowden D.W. (2011). Resequencing and analysis of variation in the tcf7l2 gene in African Americans suggests that snp rs7903146 is the causal diabetes susceptibility variant. Diabetes.

[156] Ortega-Azorin C., Sorli J.V., Estruch R., Asensio E.M., Coltell O., Gonzalez J.I., Martinez-Gonzalez M.A., Ros E., Salas-Salvado J., Fito M. (2014). Amino acid change in the carbohydrate response element binding protein is associated with lower triglycerides and myocardial infarction incidence depending on level of adherence to the mediterranean diet in the predimed trial. Circ. Cardiovasc. Genet..

[157] Castaner O., Corella D., Covas M.I., Sorli J.V., Subirana I., Flores-Mateo G., Nonell L., Bullo M., de la Torre R., Portoles O. (2013). In vivo transcriptomic profile after a mediterranean diet in high-cardiovascular risk patients: A randomized controlled trial. Am. J. Clin. Nutr..

[158] Chen Q., Turban S., Miller E.R., Appel L.J. (2012). The effects of dietary patterns on plasma renin activity: Results from the dietary approaches to stop hypertension trial. J. Hum. Hypertens..

[159] Naslund T., Silberstein D.J., Merrell W.J., Nadeau J.H., Wood A.J. (1990). Low sodium intake corrects abnormality in beta-receptor-mediated arterial vasodilation in patients with hypertension: Correlation with beta-receptor function *in vitro*. Clin. Pharmacol. Ther..

[160] Kopp U.C., DiBona G.F. (1986). Interaction between epinephrine and renal nerves in control of renin secretion rate. Am. J. Physiol..

[161] Sun B., Williams J.S., Svetkey L.P., Kolatkar N.S., Conlin P.R. (2010). Beta2-adrenergic receptor genotype affects the renin-angiotensin-aldosterone system response to the dietary approaches to stop hypertension (DASH) dietary pattern. Am. J. Clin. Nutr..

